# The ecology, evolution, and physiology of *Cardinium*: a widespread heritable endosymbiont of invertebrates

**DOI:** 10.1093/femsre/fuaf031

**Published:** 2025-07-24

**Authors:** Olivia L Mathieson, Dylan L Schultz, Martha S Hunter, Manuel Kleiner, Stephan Schmitz-Esser, Matthew R Doremus

**Affiliations:** Department of Plant and Microbial Biology, North Carolina State University, Raleigh, NC 27695, United States; Department of Animal Science, Iowa State University, Ames, IA 50011, United States; Interdepartmental Microbiology Graduate Program, Iowa State University, Ames, IA 50011, United States; Department of Entomology, The University of Arizona, Tucson, AZ 85721, United States; Department of Plant and Microbial Biology, North Carolina State University, Raleigh, NC 27695, United States; Department of Animal Science, Iowa State University, Ames, IA 50011, United States; Department of Entomology, University of Kentucky, Lexington, KY 40506, United States; Department of Entomology, University of Illinois, Urbana, IL 61801, United States

**Keywords:** *Cardinium*, heritable symbiosis, reproductive manipulation, invertebrates, arthropods, cytoplasmic incompatibility, *Wolbachia*

## Abstract

*Candidatus* Cardinium hertigii (*Cardinium*) are maternally transmitted obligate intracellular bacteria found in a wide range of invertebrate hosts, including arthropods and nematodes. Infection with *Cardinium* has substantial consequences for host biology, with many strains manipulating host reproduction to favor symbiont transmission by (i) feminizing male hosts, (ii) altering host sex allocation, (iii) inducing parthenogenesis, or (iv) causing cytoplasmic incompatibility. Other *Cardinium* strains can confer benefits to their host or alter host behavior. *Cardinium*-modified host phenotypes can result in selective sweeps of cytological elements through host populations and potentially reinforce host speciation. *Cardinium* has potential for applications in controlling arthropod pest species and arthropod-vectored disease transmission, although much remains to be explored regarding *Cardinium* physiology and host interactions. In this review, we provide an overview of *Cardinium* evolution and host distribution. We describe the various host phenotypes associated with *Cardinium* and how biological and environmental factors influence these symbioses. We also provide an overview of *Cardinium* metabolism, physiology, and potential mechanisms for interactions with hosts based on recent studies using genomics and transcriptomics. Finally, we discuss new methodologies and directions for *Cardinium* research, including improving our understanding of *Cardinium* physiology, response to environmental stress, and potential for controlling arthropod pest populations.

## Teaser

In this review, the authors synthesize nearly 30 years of research on the widespread endosymbiont *Cardinium* and its impressive effects on invertebrate biology and evolution.

## Introduction

Bacterial endosymbionts are commonly associated with terrestrial arthropods, as well as other invertebrate lineages. Many of these bacteria rely on their host for survival (i.e. obligate host-association) and use host reproduction to spread from female hosts to offspring (i.e. maternal transmission). Symbionts have evolved many mechanisms for ensuring stable transmission, including provisioning essential nutrients to hosts with nutritionally limited diets (Moran et al. 2008), protecting their host from important natural enemies or abiotic stress factors (Corbin et al. 2017, Oliver and Martinez 2014), and/or manipulating host reproduction in ways that favor infected females and improve symbiont transmission, sometimes at the expense of male hosts or uninfected females (Doremus and Hunter 2020, Werren et al. 2008). Symbionts that influence host reproduction are particularly widespread and have garnered considerable research attention given their roles in shaping arthropod evolution and speciation (Brucker and Bordenstein 2012, Gebiola et al. 2016a, Leclercq et al. 2016) and their potential for controlling arthropod populations (Gong et al. 2020, Hoffmann et al. 2011, Zheng et al. 2019).

Several bacterial lineages have evolved the ability to control and influence host reproduction. The overall phenotypes generated by these “reproductive manipulators” are very similar; however, in some cases the underlying mechanisms are assumed and have been in part shown to be completely different (Ferree et al. 2008, Harumoto et al. 2018, Harumoto and Lemaitre 2018, Penz et al. 2012, Pollmann et al. 2022). There are currently eight lineages of bacteria known to engage in reproductive manipulation, with the Alphaproteobacterium *Wolbachia* being the most widespread and most extensively researched [for recent reviews on *Wolbachia* see (Ross et al. 2019, Kaur et al. 2021, Hochstrasser 2023)]. In this review, we focus on another widely distributed endosymbiont that frequently engages in reproductive manipulation, *Candidatus* Cardinium hertigii (hereafter “*Cardinium*”). *Cardinium* is a common heritable endosymbiont found in an estimated 6%–13% of arthropod species, including arachnids, insects, and nonmarine crustaceans, as well as other invertebrates such as plant–parasitic nematodes (Duron et al. 2008a, Nakamura et al. 2009, Schön et al. 2019, Tarlachkov et al. 2023, Weinert et al. 2015, Zchori-Fein and Perlman 2004). *Cardinium* can have variable effects on hosts, but this symbiont often modifies host reproduction through several mechanisms (Doremus and Hunter 2020). These mechanisms include feminizing genetic males to develop as phenotypic females (Groot and Breeuwer 2006, Weeks et al. 2003), induction of asexual reproduction via parthenogenesis (PI), (Giorgini et al. 2009, Matalon et al. 2007, Zchori-Fein et al. 2001, 2004), and cytoplasmic incompatibility (CI) (Gebiola et al. 2016b, Gotoh et al. 2007, Hunter et al. 2003, Nakamura et al. 2012, Nguyen et al. 2017, Zhang et al. 2012). Beyond reproductive manipulation, *Cardinium* strains can alter other aspects of host biology, including host sex allocation to favor female offspring (Katlav et al. 2022a, b), oviposition (Kenyon and Hunter 2007), and feeding behavior (Ying et al. 2021). *Cardinium* may also provide both general fitness benefits (e.g. increased longevity and/or fecundity; Wang et al. 2008, Xie et al. 2016), and more conditional benefits (e.g. increased host resistance to natural enemies; Giorgini et al. 2023). The mechanisms underlying these phenotypes are largely uncharacterized and understanding how *Cardinium* induces these phenotypes remains a priority.

## Initial naming conventions and classification

**Figure 1.**
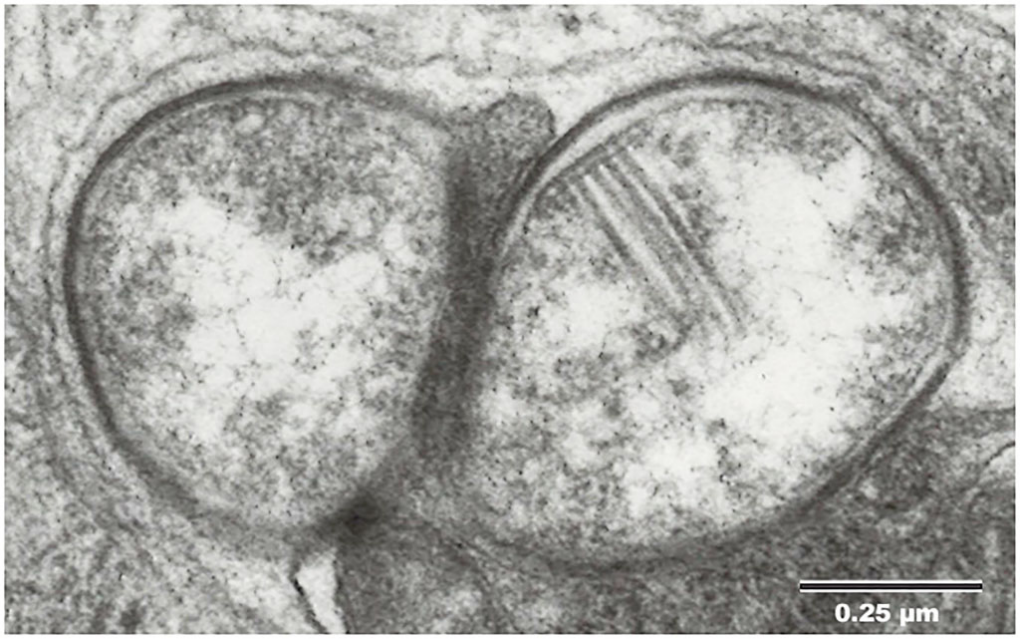
Electron micrograph of *Cardinium* in a follicle cell (surrounding the oocyte) in *Encarsia hispida*. One cell shows the parallel array of microfilament-like structures that are distinctive of *Cardinium*. The structures are hypothesized to be the intracellular components of the phage-derived type 6 secretion system T6SS^iv^ (Bock et al. 2017). Figure reproduced from Zchori-Fein et al. (2004).

The earliest reports of Gram-negative *Cytophaga*-like bacterial symbionts, later named *Cardinium*, began to emerge in the 1970s in insects (Chang and Musgrave 1972), nematodes (Shepherd et al. 1973, Walsh et al. 1983), and later in arachnids (Kurtti et al. 1996). These reports described an irregularly circular or rod-shaped Gram-negative bacterial endosymbiont belonging to the phylum Bacteroidota (previously Bacteroidetes and *Cytophaga*–*Flexibacter*–*Bacteroides*, or CFB group) with distinctive microfilament-like structures attached to the inner membrane (Fig. 1; Kurtti et al. 1996, Nakamura et al. 2009, Zchori-Fein et al. 2004). Initial naming conventions for *Cardinium* were according to its taxonomic grouping (e.g. *Cytophaga*-like; Hunter et al. 2003, Weeks et al. 2003), their hosts (e.g. *Encarsia* bacterium; Zchori-Fein et al. 2001), or some combination thereof [e.g. CFB-BP (*Brevipalpus phoenicis*); Weeks and Breeuwer 2003]. In 2004, the name “*Candidatus* Cardinium hertigii” was proposed (Zchori-Fein et al. 2004), following naming conventions for uncultured bacteria (Murray and Stackebrandt 1995). “Cardinium,” from the Latin word *cardo*, refers to the column-flanked center of Roman towns, which resemble the unique intracellular structures used to morphologically distinguish *Cardinium* from other bacterial symbionts (Fig. 1; Zchori-Fein et al. 2004). Separately, *Candidatus* Paenicardinium endonii was proposed for *Cardinium*-like symbionts found in nematodes (Noel and Atibalentja 2006). *Ca*. Paenicardinium and the *Cardinium-*like symbiont of biting midges were later collapsed into the *Ca*. Cardinium hertigii classification (Nakamura et al. 2009). Following the previous naming convention used for the endosymbiont *Wolbachia pipientis* (Werren et al. 2008), a uniform naming convention was proposed in the description of *Cardinium hertigii c*Eper1 (Penz et al. 2012). In this naming convention, *Cardinium* strains are denoted as *c*Host1, with “*c*” referring to *Cardinium*, “Host” referring to the original host that strain was identified in, and the number distinguishing unique strains co-infecting the same host system (e.g. *c*Ehis1 for *Cardinium* in *Encarsia hispida*, strain 1).

Currently, the literature differentiates *Cardinium* into at least three phylogenetic clades. Due to the broad host range and high levels of divergence between some *Cardinium* strains, it has been suggested that *Cardinium* clades should be considered distinct species rather than be collapsed under the same name (Siozios et al. 2019). Clade A has the widest range of hosts, including *Cardinium* of both mandibulate and chelicerate arthropods (Nakamura et al. 2009). *Cardinium* of nematodes make up the proposed clade B (Brown et al. 2018, Guo et al. 2022, Nakamura et al. 2009, Noel and Atibalentja 2006), with strains found in the nematode *Pratylenchus penetrans* potentially forming a separate clade (Tarlachkov et al. 2023). Clade C is composed specifically of *Cardinium* strains associated with *Culicoides* biting midges (Nakamura et al. 2009, Siozios et al. 2019). This phylogenetic structure is labile, with recently proposed phylogenies suggesting as many as seven possible clades accounting for newly identified *Cardinium* strains (Tarlachkov et al. 2023). For example, a clade including *Cardinium* of Opiliones (Chang et al. 2010, Stouthamer et al. 2019) as well as clades of *Cardinium* of freshwater copepods (Edlund et al. 2012) and ostracods (Çelen et al. 2019, Schön et al. 2019) may emerge through further work. Putative *Cardinium*-like symbionts have also been detected in the muscular foot of freshwater mollusks (Mioduchowska et al. 2020) and may represent an additional clade (Tarlachkov et al. 2023). Only limited instances of possible marine *Cardinium* have been reported in the literature to date (e.g. a bacterium found in isopods with 98% identity to *Cardinium* 16S rRNA genes; Wenzel et al. 2018).

**Figure 2.**
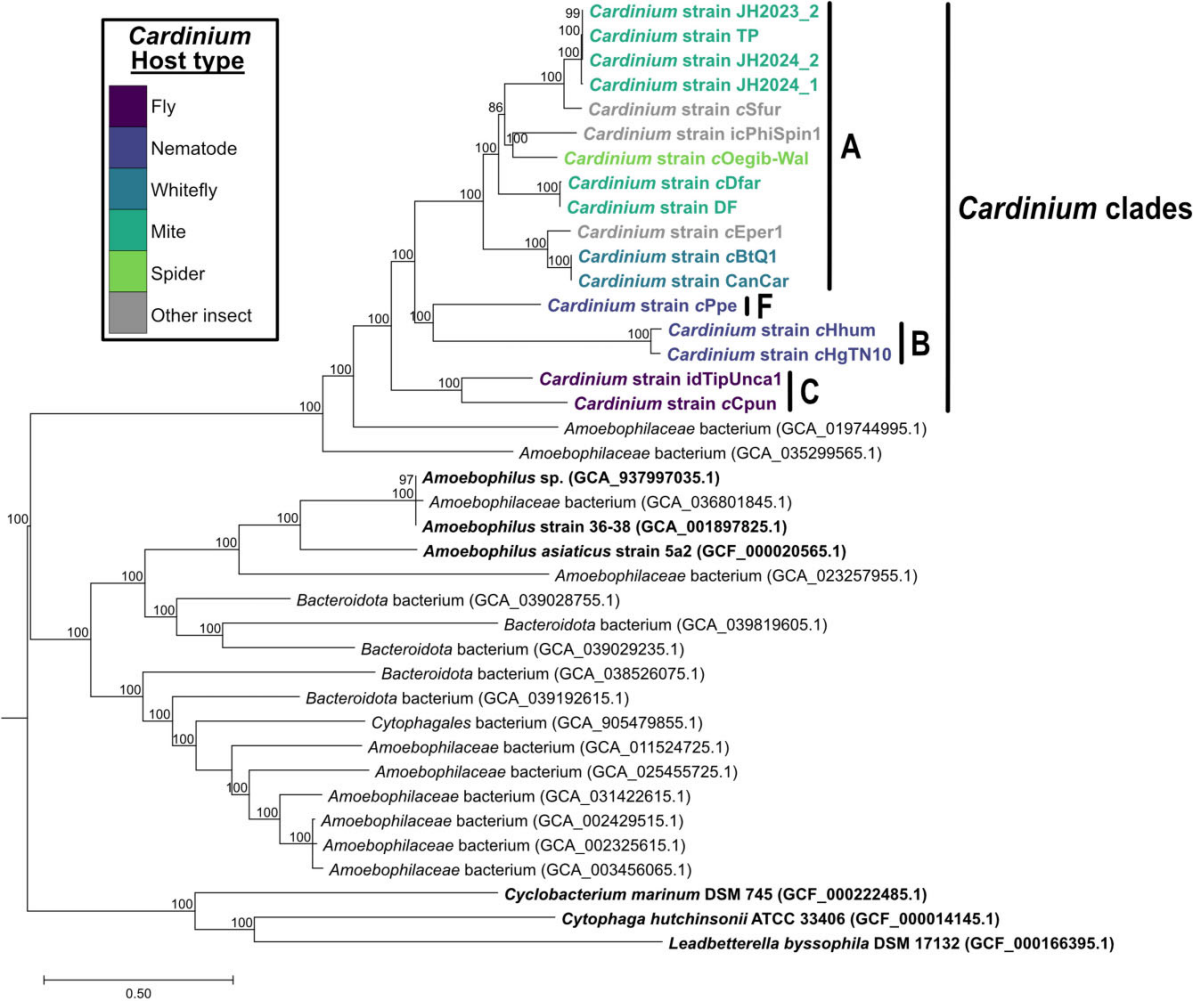
Phylogenetic tree of *Cardinium* and *Amoebophilus* genomes. Assemblies with <200 contigs classified as *Cardinium* or *Amoebophilus* (in bold) or clustering with either genus (not bolded) within the *Amoebophilaceae* family on the GTDB (Parks et al. 2022) were placed into a phylogenetic tree rooted with three free-living members of the *Cytophagales* order. The tree was constructed using the “Bacterial Genome Tree” tool on the Bacterial and Viral Bioinformatics Resource Center (BV-BRC) website with 138 single-copy genes, 5 duplications, and 5 deletions allowed (Olson et al. 2023). Briefly, amino acid and nucleotide sequences were aligned via MUSCLE (Edgar 2004) and the Codon_align function in BioPython (Cock et al. 2009), respectively, as input for tree generation with 100 rounds of rapid bootstrapping via RAxML (Stamatakis 2014, Stamatakis et al. 2008). A branch length scale bar is given on the bottom of the figure showing the estimated number of substitutions per site as an average of amino acid and nucleotide substitutions. Bootstrap confidence values above 80 are shown above each node. Proposed *Cardinium* clades supported by the tree are indicated by a black bar and clade ID (A, B, C, and F) alongside the genomes included in that clade. Colors of *Cardinium* strains correspond with their host group. Accession numbers for non-*Cardinium* genomes are given after each genome ID. Refer to Table 1 for accession numbers and other information regarding *Cardinium* genomes included in this figure.

We constructed a phylogenetic tree using 138 single-copy genes from the following set of genomes with <200 contigs: (i) assemblies classified as *Cardinium* deposited to GenBank, (ii) genomes assigned to the *Amoebophilaceae* family (which includes *Cardinium*) in the Genome Taxonomy Database (GTDB) (Parks et al. 2022), which cluster with *Cardinium* or *Candidatus* Amoebophilus asiaticus (the closest relative to *Cardinium* and a member of *Amoebophilaceae*), and (iii) three free-living organisms in the order *Cytophagales* to serve as an outgroup. The whole-genome tree supports distinct clusters for *Cardinium* and *Amoebophilus* (Fig. 2). Further, only two unclassified *Amoebophilaceae* genomes, one metagenome-assembled genome (MAG) from a soil sample (GCA_035299565.1) and one MAG from a commercial tap water sample (GCA_019744995.1), clustered with *Cardinium*, but their placement outside previously characterized clades of *Cardinium* suggests a more distant relationship to known *Cardinium* genomes. Without additional validation and host information, the taxonomic status of these genomes is unclear. *Cardinium* genomes form four main clades (Fig. 2), including a large clade A consisting of *Cardinium* hosted by mites, a spider, and various insects, clade B consisting of *Cardinium* hosted by nematodes in the genus *Heterodera*, and with *Cardinium c*Ppe hosted by the nematode *P. penetrans* forming its own clade F, congruent with what was proposed by the most recent phylogenies constructed using 16S rRNA or *gyrB* genes (Tarlachkov et al. 2023). The whole-genome tree is distinct from recent single-gene phylogenies due to the inclusion of a *Cardinium* genome from the cranefly *Tipula unca*, which was absent from prior trees. Here, it was placed into clade C with *Cardinium* hosted by *Culicoides punctatus* (midge), which previously consisted of only midge-hosted *Cardinium*. Due to the lack of assembled *Cardinium* genomes from the full range of hosts, the resolution of phylogenetic trees based on whole genomes is still limited, and other clades proposed to date cannot be assessed.

Two major contributions may enhance the resolution of the *Cardinium* phylogeny: (i) advancements in endosymbiont DNA extractions from minute hosts (Stouthamer et al. 2018), which will be particularly beneficial for hosts, such as mites and microscopic crustaceans, and (ii) the development of a multilocus sequence typing system (Stouthamer et al. 2019), which allows for efficient elucidation of *Cardinium* phylogeny using evolutionarily informative genes. Future research in the areas of *Cardinium* genomics and phylogeny should consider these tools for higher-quality genome assemblies and consistent approaches for determining evolutionary relationships.

## *Cardinium* distribution and evolution

### Distribution of *Cardinium*

*Cardinium* is an obligate endosymbiont globally distributed in a wide range of invertebrate animal hosts (Table S1), with terrestrial arthropods and nematodes the most commonly reported hosts (Nakamura et al. 2009, Tarlachkov et al. 2023). While an early estimate of *Cardinium* frequency was 6%–7% of arthropod species (Weeks et al. 2003), a more recent estimate suggests ~13% of arthropods are infected with *Cardinium*, including 60% of chelicerates and 8% of hexapods (Weinert et al. 2015). A recent microbiome survey identified *Cardinium* sequences from freshwater mollusks, marking the first instance of *Cardinium* found in hosts other than arthropods and nematodes (Mioduchowska et al. 2020). Much of what is known about *Cardinium* host range comes from field surveys of invertebrates using 16S rRNA gene-based microbial community profiling (Table S1), so it is likely that current estimates of host range and prevalence are conservative. Published estimates also do not include surveys of other host phyla such as Mollusca and Nematoda.

### Evolutionary dynamics of *Cardinium*

The sister group of *Cardinium* is *Candidatus* Amoebophilus asiaticus, a symbiont of amoebae (Horn et al. 2001, Schmitz-Esser et al. 2010). It is possible that early *Cardinium* were also symbionts of amoebae or other protists in an aquatic environment, a lifestyle which could have led to the movement of the symbiont into diverse animal hosts (Tarlachkov et al. 2023). As a maternally transmitted symbiont, the phylogenetic relationships between *Cardinium* strains can mirror those of its hosts in some mites, spiders, and Opiliones (Stouthamer et al. 2019). In some cases, these parallel phylogenetic patterns have been used to elucidate evolutionary hypotheses surrounding host divergence events (Kopecky et al. 2013). The phylogenies of *Cardinium* and its hosts are, however, not always congruent, and there is evidence of host switching events in some *Cardinium* lineages. The host diversity in clade A, for example, shows closely related pairs of *Cardinium* taxa found in parasitoid wasps (insects that lay their eggs in and consume their host) and their herbivorous insect hosts (Stouthamer et al. 2019, Tarlachkov et al. 2023, Zchori-Fein and Perlman 2004). It is unclear how often host-switching events occur or if they are evidence of more frequently occurring horizontal transmission. Some proposed mechanisms of horizontal transmission allowing for the observed host-switching include predation (Tarlachkov et al. 2023) and plant-mediated transmission (Chrostek et al. 2017, Gonella et al. 2015, Tarlachkov et al. 2023). If these alternative routes of transmission are indeed found to be prevalent, it is important that future screening measures consider possible differences between transient *Cardinium* infections (e.g. short-term presence in the gut) and long-term *Cardinium* symbioses. If possible, such studies on *Cardinium* prevalence should follow up 16S rRNA-based identifications with additional methods of confirmation such as fluorescence *in situ* hybridization, electron microscopy, or *Cardinium*-specific polyermase chain reactions (PCR) or quantitative PCR (qPCR) primers (e.g. *gyrB, sufB*, EF-G, or *groEL*) to achieve higher confidence of identification [see Stouthamer et al. (2019) for suggested primers].

*Cardinium* strains cause different host phenotypes, including several forms of reproductive manipulation (discussed below). Strains inducing similar effects often do not form monophyletic groups, suggesting that if the common ancestor of *Cardinium* had factors for multiple reproductive manipulations, these manipulative factors were subsequently lost or inactivated as *Cardinium* diversified and adapted to different hosts (Stouthamer et al. 2019). Alternatively, factors responsible for these phenotypes may have arose through convergent evolution and/or horizontal gene transfer between distantly related symbiont lineages (Stouthamer et al. 2019). While intracellular endosymbionts like *Cardinium* do not encounter other bacterial species as frequently as extracellular symbionts, instances of *Cardinium* cooccurring with other intracellular symbionts like *Wolbachia* in the same host have been observed in several insect, arachnid, and nematode groups (e.g. Brown et al. 2018, Konecka and Olszanowski 2019, Ros et al. 2012, Ros and Breeuwer 2009, White et al. 2009, Xie et al. 2016, Zhang et al. 2012, Zhu et al. 2012). These symbiont co-infections could potentially provide an opportunity for intracellular heritable endosymbionts to exchange genetic material via horizontal gene transfer, which may have played an important role in shaping the accessory functions in *Cardinium* genomes (Brown et al. 2018, Siozios et al. 2019). There is also the possibility of interkingdom horizontal gene transfer events between intracellular symbionts and their eukaryotic hosts (Hotopp et al. 2007, Leclercq et al. 2016). However, no such occurrences have been reported in *Cardinium* symbioses to date. Host biology may also have some influence on phenotype induction, as symbiont strains transinfected between species can cause different host phenotypes (Li et al. 2020a). Whether *Cardinium* had the ability to manipulate eukaryotic reproduction ancestrally or if *Cardinium* strains acquired their manipulative capabilities through convergent evolution or horizontal gene transfer remains an open question. Resolving this will require further mechanistic characterizations in multiple host species to identify factors responsible for these phenotypes, followed by analyses into the distribution of manipulative factors among *Cardinium* strains.

## Phenotypes associated with *Cardinium* infection

**Figure 3.**
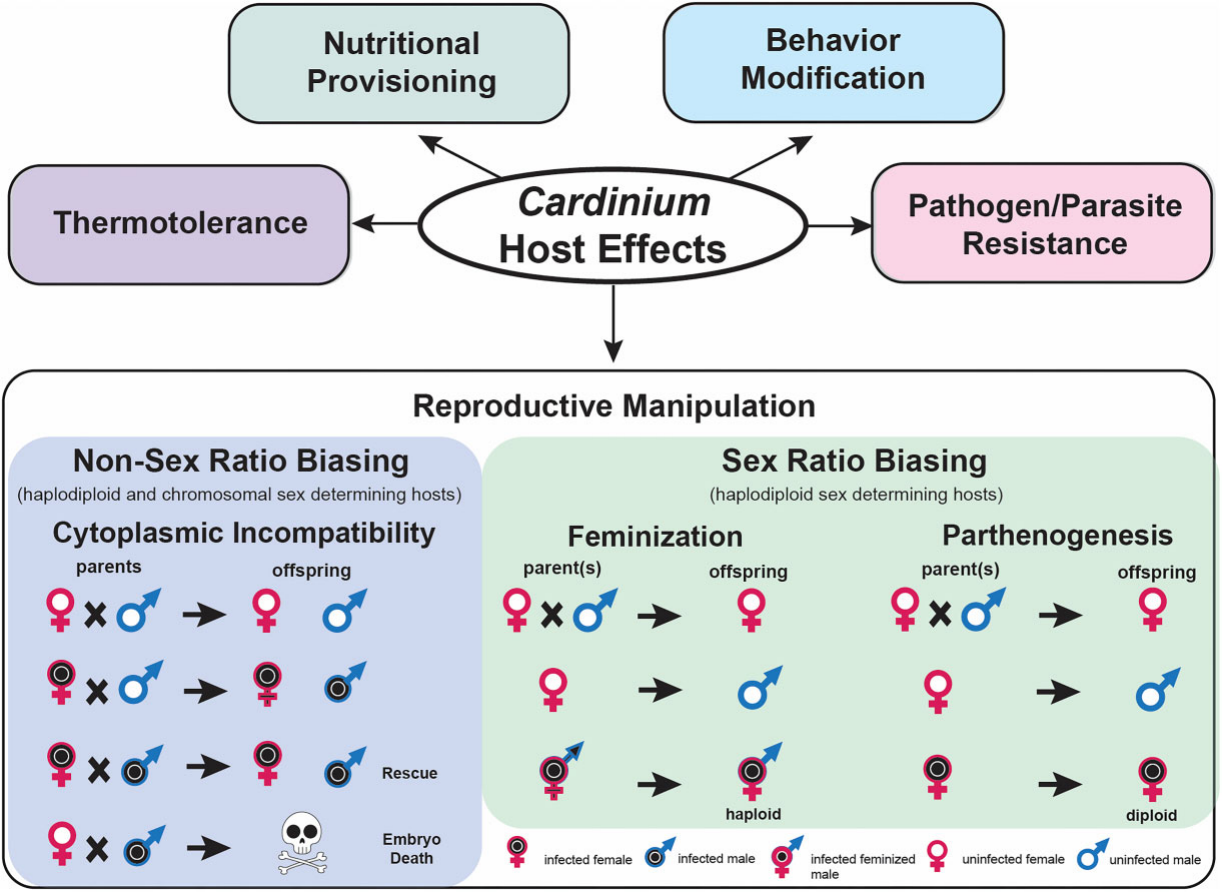
Phenotypes associated with *Cardinium* infection. Known forms of reproductive manipulation induced by *Cardinium* include CI, PI, and feminization. These phenotypes facilitate *Cardinium* transmission by providing a fitness advantage to infected females at the expense of uninfected females (CI) or by skewing reproduction to favor female progeny (PI, feminization). CI is a two-step process that drives the infection through a population: first, *Cardinium* sabotages male hosts causing offspring death when that male mates with uninfected females. Second, *Cardinium* prevents this modification from killing infected offspring. PI *Cardinium* cause female hosts to asexually produce other females, while feminizing *Cardinium* cause genetic males to develop as functional females. Feminizing *Cardinium* have best been described in haplodiploid *Brevipalpus* mites, which asexually produce haploid male offspring; *Cardinium* feminizes these haploid males, resulting in an asexual population of haploid females (Weeks et al. 2001, Groot and Breeuwer 2006). Other possible phenotypes associated with *Cardinium* infection include thermotolerance, nutritional provisioning (in cosymbiosis), behavior modifications, and protection from natural enemies and pathogens.

*Cardinium* can cause a variety of phenotypes in its hosts (Fig. 3). Some phenotypes manifest via modifications to host reproduction. These phenotypes benefit *Cardinium* by skewing host reproduction to favor the number or fitness of infected females that transmit the symbiont and may impose a cost to uninfected females or male hosts. This control of host reproduction by symbionts like *Cardinium* is referred to as reproductive manipulation. Additionally, some *Cardinium* confer conditionally dependent benefits to their hosts, increasing host fitness and their own fitness alike under particular environments or abiotic conditions. The reproductive manipulation cytoplasmic incompatibility (CI) is commonly associated with *Cardinium*. CI is a lethal manipulation of infected males that kills uninfected offspring. Other reproductive manipulator phenotypes bias reproduction to favor infected females which transmit *Cardinium*. To attribute a phenotype to heritable symbionts like *Cardinium*, a set of criteria have been established and followed in the research community (Stouthamer et al. 1999). The criteria for confirming *Cardinium*-induced phenotypes are: (i) The phenotype is present only when *Cardinium* is present and is absent in hosts where the symbiont has been removed. Additionally, for putative parthenogenesis-inducing symbionts, males are produced by uninfected hosts. (ii) *Cardinium* is the only heritable symbiont present in the host with the phenotype. Differential offspring production or offspring sex ratios between infected and uninfected hosts are suggestive of CI or parthenogenesis and feminization, respectively (Hunter et al. 2003, Zchori-Fein et al. 2004). In this section, we discuss the major phenotypes attributed to *Cardinium* infection, including how *Cardinium* induces these phenotypes and the biological and environmental factors that influence their efficacy (Fig. 3).

### Host reproductive manipulation via CI

**Figure 4.**
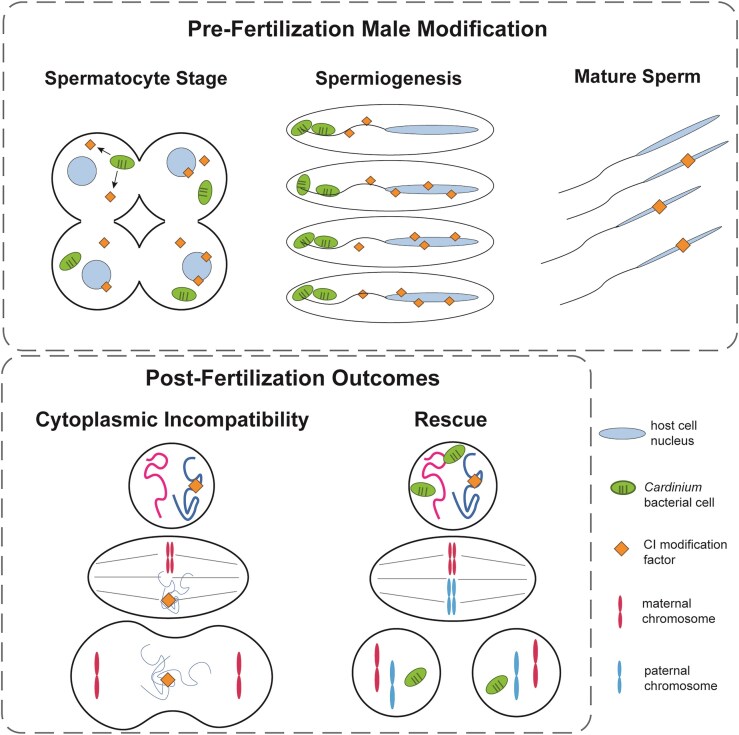
Hypothetical mechanism of *Cardinium*-induced CI and rescue based on cytology and fluorescence microscopy of *Encarsia suzannae* testes and embryos. *Cardinium*-induced CI in *E. suzannae* is initiated during spermatogenesis, possibly by altering male chromatin or DNA-associated proteins, or by loading sperm with a toxin that is released upon fertilization. The second scenario is illustrated here. Either scenario causes asynchronous chromosomal segregation during mitosis in early embryogenesis that kills uninfected offspring. To rescue CI, *Cardinium* may reverse the alterations made to male chromatin or similarly alter female chromatin to restore mitotic synchrony or produce an antitoxin to prevent the sperm-delivered toxin from killing the embryo.

The phenotype most widely associated with *Cardinium* infection is CI, with CI *Cardinium* strains observed in parasitoid wasps (Gebiola et al. 2016b, Hunter et al. 2003), planthoppers (Nakamura et al. 2012), thrips (Nguyen et al. 2017), and mites (Gotoh et al. 2007, Ros and Breeuwer 2009, Xie et al. 2016, Zhu et al. 2012). CI is a two-step lethal manipulation composed of a modification step in males and a rescue step in eggs (Fig. 4). During modification, the symbiont first sabotages male sperm or other paternal products to kill their offspring during embryogenesis. Next, the maternally transmitted symbiont “rescues” infected embryos by nullifying the toxicity of modified sperm and restoring the viability of rescued embryos; uninfected females’ embryos are not rescued and die early in development. Together, the modification and rescue steps of CI grant a fitness advantage to infected females since they produce viable infected offspring with both infected and uninfected mates. This drives symbiont infections to high frequencies in most host populations and promotes stable transmission of the symbiont (Harris et al. 2010, Hoffmann and Turelli 1997). CI can also greatly influence host evolution by contributing to host speciation, with symbionts acting as reproductive barriers (Brucker and Bordenstein 2012, Gebiola et al. 2016a) and causing selective sweeps in host populations as CI symbionts drive themselves through a population (Hurst and Jiggins 2005).

Due to their ability to drive themselves and associated cytoplasmic elements through a population, CI symbionts also have promising applications for controlling the transmission of arthropod-vectored pathogens if the drive provided by CI symbionts can be paired with other desirable traits like host pathogen resistance (Gong et al. 2020, Hoffmann et al. 2011, Moreira et al. 2009, Zheng et al. 2019). This is exemplified by the successful suppression of Dengue transmission in Australia following release of CI *Wolbachia*-infected *Aedes aegypti: Wolbachia* alone increases *A. aegypti* resistance to dengue infection along with inducing CI to drive the infection and pathogen resistance through natural populations (Hoffmann et al. 2011, Moreira et al. 2009, Ryan et al. 2020). It is currently unknown whether *Cardinium* can also confer host resistance to vector-borne diseases, but as *Cardinium* research advances, novel host impacts may be revealed. The evolutionary ramifications of symbiont-induced CI, as well as the potential application of CI symbionts in the control of pest populations and vector-borne diseases, have spurred sustained interest in the cellular and genetic mechanisms underlying CI.

Recent work on the cellular localization of two CI *Cardinium* strains in male reproductive tissues of *Encarsia* parasitoid wasps has provided some insight into how *Cardinium* induces CI in its hosts (Doremus et al. 2022, 2020). In *Encarsia suzannae*, the *Cardinium* strain *c*Eper1 densely infects most developing sperm cells during pupation, the life stage critical to CI modification in this host. *Cardinium* cells are removed from developing sperm during spermiogenesis, the final stage of sperm development associated with large-scale morphological and nuclear restructuring of sperm cells (Ferree et al. 2019). The loss of *c*Eper1 coincides with the onset of sperm maturity and the end of the CI modification window, suggesting that *c*Eper1 modifies developing sperm cells internally to induce CI prior to its removal during spermiogenesis (Doremus et al. 2020). This localization pattern is similar to that of some CI *Wolbachia* strains, including *w*Mel and *w*Ri, which infect *Drosophila* (Clark et al. 2003, 2002) as well as CI *Wolbachia* that infect the wasp *Nasonia vitripennis* (Clark et al. 2008). Both *Cardinium* and *Wolbachia* infect developing sperm cells and seemingly modify these cells prior to symbiont removal in spermiogenesis.

A second CI *Cardinium* strain, *c*Eina3, shows a strikingly different infection density and localization pattern in the male reproductive system of the wasp *Encarsia partenopea* (Doremus et al. 2022). Despite apparently few cells infecting the male reproductive system, *c*Eina3 causes a more consistently lethal CI phenotype than *c*Eper1. This low-density *c*Eina3 infection is also associated with a different infection pattern: instead of infecting sperm cells, most *c*Eina3 cells infect somatic cells at the base of the testis and around the seminal vesicle, where mature sperm cells are stored. This unique localization pattern suggests that *c*Eina3 uses a different modification method and/or set of CI effectors to indirectly modify either developing sperm cells within the testes or fully mature sperm within the seminal vesicle (Doremus et al. 2022). It is possible that this second type of localization pattern contributes to the more lethal CI phenotype associated with *c*Eina3, although variation in CI effectors and their expression, as well as host factors could also contribute to the observed differences in CI strength. Regardless of the underlying mechanism, CI-inducing symbionts can display a variety of localization patterns in the tissues of closely related hosts. Characterization of the localization patterns of other CI *Cardinium* strains could help determine the extent of this variation, whether localization correlates with CI lethality and CI effector identity, and which host tissues are relevant to CI induction.

The cytological defects arising from *Cardinium* CI were first characterized for the *c*Eper1 strain in *E. suzannae*. Following fertilization, CI-affected embryos display several mitotic defects including abnormal chromatin condensation and chromatin segregation that can produce chromatin bridges between daughter nuclei. These mitotic defects persist through multiple cleavage divisions, with the accumulation of these defects ultimately leading to late embryonic mortality (Gebiola et al. 2017a). The cytological defects caused by *c*Eper1 CI are similar to those caused by *Wolbachia* CI (Callaini et al. 1997, Tram and Sullivan 2002), although *Wolbachia* CI usually kills the embryo earlier in development than *Cardinium* CI (Callaini et al. 1996, Tram et al. 2006). These similarities between *Cardinium* and *Wolbachia* CI at the cellular level seem to be an instance of convergent evolution, as *Cardinium* genomes do not encode the *Wolbachia* CI factors (Penz et al. 2012). While genomic and transcriptomic analyses of *c*Eper1 have not yet revealed obvious candidate *Cardinium* CI factors (Mann et al. 2017, Penz et al. 2012), these studies, along with a new understanding of *Cardinium* localization in male tissues during CI modification, form a basis to potentially identify *c*Eper1 proteins involved in CI. Identifying these CI factors and their host targets will provide insights into the origin of *Cardinium* CI, and reveal where the CI mechanisms of *Cardinium* and *Wolbachia* converge.

### Parthenogenesis and feminization

**Figure 5.**
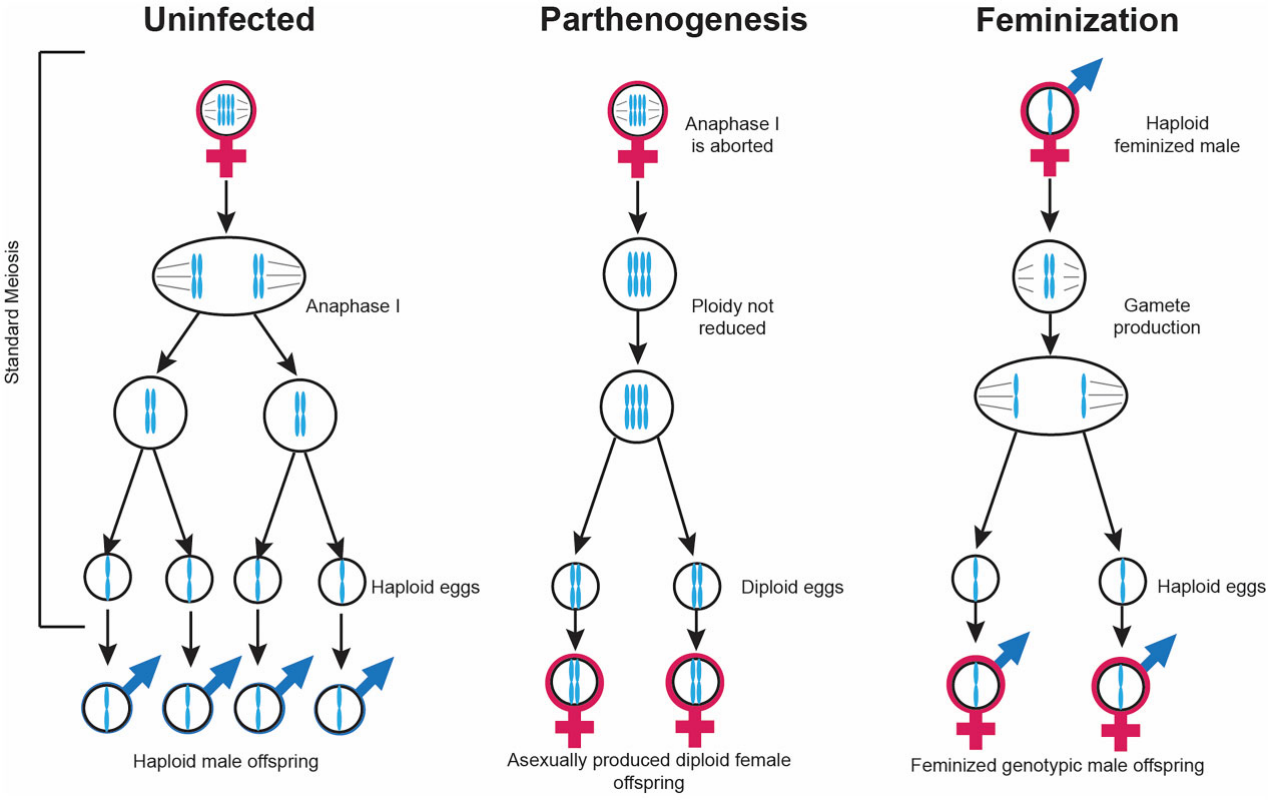
*Cardinium* feminization and parthenogenesis both manipulate the asexual production of haplodiploid hosts, turning male offspring into females. In haplodiploid hosts, unfertilized haploid eggs develop as males, while fertilized diploid eggs develop as females. The feminization model for *Cardinium* is based on *Brevipalpus* mites, where haploid unfertilized male eggs develop as functional phenotypic females (Weeks et al. 2001, Groot and Breeuwer 2006). This results in populations of haploid individuals that reproduce asexually. Parthenogenesis causes the asexual production of female offspring via a two-step process in *E. hispida. Cardinium* first inhibits offspring ploidy reduction by disrupting the anaphase 1 step of meiosis, then feminizes diploid eggs to develop as females (Giorgini et al. 2007, 2009).

*Cardinium* is commonly associated with two phenotypes that result in female-biased sex ratios: parthenogenesis induction (PI) and feminization. PI enables infected females to asexually produce more infected females, eventually resulting in an entirely female asexual host population (Fig. 5). In insects, *Cardinium*-induced PI has only been demonstrated in hosts exhibiting haplodiploidy, in which diploid females can produce haploid male offspring asexually while females develop from fertilized diploid embryos (Matalon et al. 2007, Provencher et al. 2005, Zchori-Fein et al. 2001, 2004). In haplodiploid hosts, PI *Cardinium* cause diploid females to asexually produce diploid daughters. More recently, *Cardinium* has also been associated with PI in nonmarine ostracods, whose ploidy status is unclear (Schön and Martens 2020).

*Cardinium* has been associated with parthenogenesis in several lineages of hosts, including parasitoid wasps (Matalon et al. 2007, Zchori-Fein et al. 2001, 2004), armored scale insects (Provencher et al. 2005), and nonmarine ostracods (Schön and Martens 2020). Unfortunately, symbiont-induced PI is difficult to confirm in many cases, as parthenogenetic hosts often lose the ability to reproduce sexually and PI-associated symbionts can become essential for reproduction, limiting the potential for experimental manipulation of the symbiont (Fricke and Lindsey 2024a, Stouthamer et al. 2010, Zchori-Fein et al. 2001). In most instances, the role of *Cardinium* in host parthenogenesis has not been confirmed and is instead correlated with the presence of *Cardinium* in asexual, but not sexual species (Provencher et al. 2005, Schön and Martens 2020, Zchori-Fein et al. 2001). This is the case for the parthenogenetic wasp *Encarsia tabicivora* (formerly classified as *Encarsia pergandiella*; Gebiola et al. 2017b), in which *Cardinium* is associated with parthenogenesis but is also essential for successful wasp reproduction (Zchori-Fein et al. 2001). The role of *Cardinium* as the causal agent of PI has been confirmed by symbiosis disruption via antibiotics or heat in only two parasitoid wasp species, *Plagiomerus diaspidis* (Gordh and Lacey 1976, Matalon et al. 2007, Zchori-Fein and Perlman 2004) and *Encarsia hispida* (Giorgini 2001, Giorgini et al. 2009, Zchori-Fein et al. 2004). In both cases, these wasps began asexually producing male offspring following the loss of *Cardinium*, as would be expected in a haplodiploid host (Giorgini 2001, Giorgini et al. 2009, Gordh and Lacey 1976, Zchori-Fein et al. 2004).

*Cardinium* PI has been best characterized in haplodiploid *Encarsia* wasps. In *E. hispida* and *E. tabacivora*, restoration of diploidy in unfertilized eggs is caused by the central fusion of sister chromosomes either during meiosis I or immediately following it (Doremus and Hunter 2020, Giorgini et al. 2007). This contrasts with *Wolbachia* PI, which restores diploidy via gamete duplication following the completion of meiosis (Giorgini et al. 2007). These differences in the timing and manner of diploidy restoration suggest that *Cardinium* and *Wolbachia* may have independently evolved PI, as is the case for CI. The antibiotic treatment of *E. hispida* curiously results in the production of diploid males, indicating that diploidization alone is not sufficient for PI and that *Cardinium* must additionally feminize diploid hosts to induce parthenogenesis (Giorgini et al. 2009). These results suggest that *Cardinium* uses a two-step mechanism to induce PI, composed of initial diploidization followed by feminization later in development. If PI is a two-step process, feeding adult female *E. hispida* antibiotics may have disrupted the feminization but not diploidization steps of PI in their offspring leading to the production of diploid males. This work highlights the importance of characterizing offspring ploidy following symbiosis disruption in suspected PI systems, which can indicate whether diploidy restoration is sufficient for inducing female development or if a second feminizing step is required. A two-step PI mechanism involving host feminization via manipulation of the sex determination system has also been proposed for some PI *Wolbachia* strains (Ma and Schwander 2017). Recent work identifying *Wolbachia* PI factors supports a two-factor mechanism for some *Wolbachia* strains, with one of the two factors acting as a potential mimic of *transformer*, a key insect sex determination factor (Fricke and Lindsey 2024b, Li et al. 2024a). Whether *Cardinium* PI strains use a similar mechanism to feminize hosts remains to be seen.

Feminization differs from PI by causing infected hosts that harbor the chromosomal configuration for male development to develop as fully functional, phenotypic females (Fig. 5). Unlike populations affected by PI, in which males are no longer necessary for females to reproduce, males are usually still required for reproduction in feminized populations. A curious exception is in *Brevipalpus* mites feminized by *Cardinium*, wherein genetic haploid male mites instead develop into haploid females, resulting in an entirely haploid female population that reproduces asexually (Groot and Breeuwer 2006, Weeks et al. 2001). Exposure to antibiotics results in an increased production of male offspring, confirming the role of *Cardinium* in causing female development (Groot and Breeuwer 2006). The feminization of haploid females results in a scenario functionally similar to PI, as infected females can asexually produce more feminized females (Groot and Breeuwer 2006, Weeks et al. 2001). If *Cardinium* PI is caused by two independent diploidization and feminization steps, it is possible that the *Cardinium* strains in *Brevipalpus* mites have retained the ability to feminize hosts but lost the ability to restore diploidy. Alternatively, feminizing *Cardinium* in *Brevipalpus* may use an independent method of feminization that is not analogous to the process used by PI *Cardinium* strains. Identification of PI factors involved in diploidization and feminization will help resolve whether these *Cardinium* use the same processes to induce asexual female reproduction.

While most sex-ratio distorting *Cardinium* are associated with asexual reproduction, there may be other methods by which *Cardinium* can bias host sex ratio to favor females. One such alternative method involves female-biased offspring sex allocation in the haplodiploid citrus thrips *Pezothrips kellyanus*, which is co-infected with CI-inducing strains of *Cardinium* and *Wolbachia*. Egg fertilization in *P. kellyanus* is egg size-dependent, with larger eggs preferentially fertilized to produce female offspring (Katlav et al. 2021). This results in individual females producing broods with either strongly female-biased or male-biased sex ratios. While uninfected females can display female-biased sex allocation, those hosting *Cardinium* are even more likely to produce female-biased broods, suggesting that symbiont infection biases sex allocation to favor female offspring (Katlav et al. 2022a). This sex allocation effect may in part be caused by a general increase in fitness, as *Cardinium*-infected females are larger than uninfected ones or those co-infected with *Wolbachia*. These larger *Cardinium*-infected females in turn produce larger eggs than uninfected or co-infected females, which are then preferentially fertilized (Katlav et al. 2022a). *Cardinium* seemingly affects maternal resource investment in eggs, although research on the metabolic contribution of this *Cardinium* may reveal more information on this unique phenotype. Similarly, egg size-based selective fertilization has been described in other haplodiploid arthropods, although it remains to be seen if symbionts in other systems also influence sex allocation (Macke et al. 2010).

### Other host phenotypes associated with *Cardinium* infection

*Cardinium* is also associated with a range of host phenotypes beyond reproductive manipulation. These include modifying host oviposition behavior of *Encarsia* wasps (Kenyon and Hunter 2007) and mating preferences of *P. kellyanus* (Tourani et al. 2024), the latter of which may improve the fitness of *Cardinium*-infected females through the avoidance of lethally incompatible males that have *Wolbachia. Cardinium* can also provide general beneficial effects such as increased host fecundity and higher survival rates (Katlav et al. 2022a, Wang et al. 2008, Xie et al. 2016). How *Cardinium* increases host fitness is not clear, but it may involve some degree of metabolic provisioning (Newton and Rice 2020). *Cardinium* can also be costly for the host, reducing fecundity and survival by using host nutritional resources or, in the case of some Mediterranean (MED) biotype *Bemisia* whiteflies, altering feeding behavior (Shan and Liu 2021, Ying et al. 2021). *Cardinium* may also provide more conditional benefits, although most have only been observed in whitefly hosts. Such benefits include potentially augmenting host resistance to parasitoids in MED *Bemisia* whiteflies (Giorgini et al. 2023), altering interactions between whitefly hosts and their host plants (Liu et al. 2023), or improving MED *Bemisia* whitefly thermotolerance (Yang et al. 2021). MED *Bemisia* whiteflies collected from warmer regions of China also show higher *Cardinium* infection rates, supporting a link between higher temperatures and *Cardinium* infection in these populations (Li et al. 2023a). Further characterization of the impact of *Cardinium* infection on host thermotolerance is warranted given the increasing severity of global climate change and its implications for arthropod biology.

In addition to modifying host abiotic stress tolerance, heritable endosymbionts can augment host immunity via providing protection from natural enemies and pathogens (Oliver and Martinez 2014). While some symbionts protect their host by producing specific toxins targeting parasites or pathogens (Degnan and Moran 2008, Hamilton et al. 2016), other symbionts, most notably *Wolbachia*, may provide more general protection by competing with pathogens or modulating the host immune response (Rances et al. 2012, Caragata et al. 2013, Bhattacharya et al. 2017). The role of *Cardinium* in host immunity presents a clear gap in our understanding of *Cardinium* biology, particularly given the potential for endosymbionts to help control arthropod-vectored disease transmission exemplified by the World Mosquito Program (O’Neill 2018). A study comparing the responses of a lepidopteran cell line to *Cardinium* infection found that *Cardinium* elicited an upregulated immune response, suggesting some degree of interplay between *Cardinium* and insect immunity (Nakamura et al. 2011). Whether this upregulated immune response in an *in vitro* cell line translates to *in vivo* effects in natural hosts has not been thoroughly explored. In addition to potentially upregulating host immune responses to parasites and pathogens (Kambris et al. 2010), these interactions between *Cardinium* and the host immune system could also play roles in regulating these symbioses and may impede the establishment of new symbioses (Weinert et al. 2015). One hindrance to studying the defensive benefits of *Cardinium* has been the lack of known natural enemies or pathogens of the more extensively studied *Cardinium* hosts (e.g. *Encarsia* wasps and *Brevipalpus* mites). Yet, *Cardinium* is also present in certain agricultural and medical pests like planthoppers, whiteflies, and biting midges, providing a promising opportunity to study how *Cardinium* interacts with anthropogenically relevant insect-vectored pathogens (Gilbertson et al. 2015, Mellor et al. 2000, Zhou et al. 2008).

While *Cardinium-*associated phenotypes have been explored in a number of different arthropod hosts, almost nothing is known of the phenotypic consequences of *Cardinium* infection in nematodes, aquatic crustaceans, or spiders, the latter of which appear particularly prone to hosting *Cardinium* (Duron et al. 2008b). In *P. penetrans*, a plant–parasitic nematode co-infected with *Cardinium* and *Wolbachia, Wolbachia* was found to be associated with a female-biased sex ratio, although it is unclear whether *Cardinium* is a reproductive manipulator in this host as well (Brown et al. 2018, Wasala et al. 2019). *Cardinium* and *Wolbachia* may also participate in metabolic complementation for the biosynthesis of methionine or fatty acids in *P. penetrans* (Brown et al. 2018). In nonmarine ostracods, *Cardinium* infection is associated with parthenogenetic populations (Schön et al. 2019, Schön and Martens 2020). As this association was only recently identified, *Cardinium* has not yet been confirmed as the causal agent of parthenogenesis. It is likely that we currently underestimate both the prevalence of *Cardinium* in nematode and aquatic systems and its phenotypic impact on these hosts. The presence of manipulative symbionts in aquatic systems is particularly interesting, given that the terrestrial models for reproductive manipulation may not hold true in marine environments, where broadcast spawning is a prevalent reproductive strategy (Kustra and Carrier 2022). New approaches may be required for identifying reproductive manipulation phenotypes in such host groups (Kustra and Carrier 2022).

As with many obligate host-associated symbionts, most of what is known about *Cardinium* comes from genomic information and studies on infected insect cultures since direct culturing of the bacterium is currently not possible. However, successful efforts to maintain *Cardinium* in arthropod cell lines provide a promising avenue for exploring phenotypic effects on the cellular level. The initial isolation of *Cardinium* in an *Ixodes* tick cell line (Kurtti et al. 1996) was followed by successful propagation of *Cardinium* in mosquito cell lines (AeAI-2 and C7-10) and in a lepidopteran cell line (HZ-AM1) (Morimoto et al. 2006). The *Ixodes*-derived *Cardinium* were also propagated in silkworm cells (Bm-aff3) (Nakamura et al. 2011). Successful propagation of *Cardinium* derived from host lineages besides *Ixodes* ticks has not been reported, to date.

### Factors affecting *Cardinium* symbioses and phenotypes

Numerous biotic and abiotic factors influence the stability of heritable symbioses and the penetrance of symbiont-induced phenotypes, such as host factors, symbiont titer, co-infecting symbionts, or external environmental stressors. These effects may also be mediated by genotype differences among symbiont and host lineages, although the influence of host genotype on *Cardinium*-induced phenotypes has not been widely explored.

Host genotype interactions with *Cardinium* symbioses have been most extensively studied in the MED biotype *Bemisia tabaci* whiteflies, with the fitness benefits of *Cardinium* infection, including increased fecundity, decreased developmental time, and increased longevity, varying between different whitefly lineages (Li et al. 2023b). The influence of host lineage also extends to *Cardinium*-conferred thermotolerance, with only certain whitefly lineages benefiting from *Cardinium* infection following heat exposure (Yang et al. 2021). Experimental transinfection of *Cardinium* between host species also suggests that host genotype in part determines the phenotypic effects of *Cardinium*. A CI-inducing *Cardinium* strain was successfully transferred between two planthopper species. While this *Cardinium* strain caused CI in its native host, it did not induce CI in the novel planthopper (Li et al. 2020b).

Other host factors, like host age, can also modify the manifestation of *Cardinium*-induced phenotypes. *Cardinium* CI lethality decreases as male adults age in the planthopper *Sogatella furcifera* and the carmine spider mite *Tetranychus cinnabarinus* (Nakamura et al. 2012, Noda et al. 2001, Xie et al. 2010). Yet, in the wasp *E. suzannae*, adult male age does not affect *Cardinium* CI; instead, male pupal developmental time is the most important factor for determining *Cardinium* CI strength in this wasp (Doremus et al. 2019, Perlman et al. 2014). This is likely due to the timing of sperm production and CI male modification in *E. suzannae*, both of which are completed primarily during pupation (Doremus et al. 2020). Host age increases feminization efficacy in *Cardinium*-infected *Brevipalpus* mites, with the production of male offspring rapidly decreasing as adult females age. In this case, *Cardinium* titer may increase as females age, resulting in a greater proportion of eggs undergoing successful feminization in older females (Groot and Breeuwer 2006). Many of these differences observed between *Cardinium* symbioses likely arise from idiosyncrasies of host biology.

The influence of *Cardinium* titer in determining phenotype expression also varies depending on the symbiosis. In the carmine spider mite, *T. cinnabarinus, Cardinium* CI lethality is tightly associated with symbiont titer, with a reduction in titer resulting in weaker CI (Xie et al. 2010). However, in *E. suzannae* and the mite *Tetranychus urticae*, reduction in *Cardinium* titer does not always correspond with reduced CI (Doremus et al. 2019, Xie et al. 2016). As discussed above, in the wasp *E. partenopea*, a low titer *Cardinium* strain is capable of inducing uniformly lethal CI, further indicating that some *Cardinium* strains can consistently impose phenotypes upon the host despite their low density (Doremus et al. 2022). Why titer affects some *Cardinium* symbioses more than others remains an open question, but likely involves differences in *Cardinium*-encoded factors, gene expression, localization, and/or host susceptibility to manipulation.

Heritable symbionts can share their host with other co-infecting symbiont lineages. These symbiont co-infections offer an opportunity for host-restricted symbiont lineages to interact with one another, often with consequences that extend to the host. *Cardinium* is frequently found co-infecting hosts with other manipulative symbionts like *Wolbachia* (Hubert et al. 2021b, Nakamura et al. 2012, Nguyen et al. 2017, White et al. 2011). The outcomes of these co-infections seem to be variable, ranging from additive (Nakamura et al. 2012, Nguyen et al. 2017), to neutral (White et al. 2011), or weakening effects on symbiont-induced phenotypes (Hubert et al. 2021b, Ros and Breeuwer 2009, Zhang et al. 2012, Zhao et al. 2018) and likely depend on the symbiont strains and host involved. In several cases, co-infecting *Cardinium* strains are asymptomatic, at least with regard to reproductive manipulation (Doremus et al. 2022, White et al. 2011). In these cases, the asymptomatic *Cardinium* strain co-infects hosts along with a CI-inducing symbiont (Doremus et al. 2022, White et al. 2009). How these asymptomatic *Cardinium* strains spread is unclear; because of their occurrence in coinfections, they may act as heritable hitchhikers passively spreading via the CI and/or selective advantages provided by their neighbor. These strains may also provide conditional benefits that facilitate their spread or may be former reproductive manipulators that subsequently lost their manipulative capabilities. The propensity of *Cardinium* for co-infection, along with the variable outcomes of these interactions, make it an intriguing model for studying how symbionts respond to one another, as well as the evolutionary and ecological ramifications of co-infections.

The external environment can exert a strong influence on heritable symbioses. Heritable symbionts are notorious for their susceptibility to environmental stress, particularly thermal stress, which can reduce symbiont titer, transmission rate, and phenotypic expression (Corbin et al. 2017). Yet, a survey of global symbiont infections found a positive association between *Cardinium* infection and temperature, particularly in mandibulate arthropods like true bugs, suggesting that *Cardinium* may better tolerate warmer temperatures than symbionts like *Wolbachia* (Charlesworth et al. 2019). More localized surveys of *Cardinium* infections in specific host populations have yielded similar results. A study of *Cardinium* prevalence in Chinese populations of the spider mite *T. cinnabarinus* only found *Cardinium* in host populations in milder warm and wet regions compared to more arid regions prone to daily extreme temperature fluctuations (Liu et al. 2006). A similar infection pattern was found for *Cardinium* infecting MED *B. tabaci* whiteflies in China (Li et al. 2023a) and in *Culicoides* biting midges in Israel (Morag et al. 2012), suggesting that these infection patterns may be comparable across *Cardinium* symbioses. Together these surveys suggest that extreme fluctuations in temperature may be more important than mean temperatures for limiting *Cardinium* prevalence in nature. These surveys also highlight humidity as an additional factor that may be important for *Cardinium* infection stability. Whether *Cardinium* itself is susceptible to changes in humidity (seemingly unlikely because of its intracellular habitat), or if low humidity indirectly inhibits *Cardinium* spread by compounding its fitness costs or altering the host intracellular environment is not clear. In *Culicoides* midges, an indirect effect of humidity may be more likely as these midges rely on moist environments for their development (Morag et al. 2012).

While infection surveys can provide some insight into the climatic forces shaping *Cardinium* symbioses, there are few studies directly testing the effect of environmental stressors on *Cardinium* symbioses. In some cases, like in MED *B. tabaci* whiteflies, *Cardinium* seem to respond positively to warmer temperatures, with *Cardinium* titer (Yang et al. 2023a) and whitefly survival and fecundity increasing following warm temperature exposure (Yang et al. 2021). Yet, in the whitefly parasitoid *E. suzannae*, similarly warm temperatures negatively affect the symbiosis by reducing *Cardinium* titer, transmission rate, and CI strength (Doremus et al. 2019). In citrus thrips, exposure to warm temperatures reduced the female-biasing effect of *Cardinium* but did not affect *Cardinium* CI or titer (Katlav et al. 2022b). These three studies show that substantial variation in response to temperature stress exists among *Cardinium* strains, with the caveats that these different observations could be the result of different methodological approaches (i.e. severity of temperature exposures), host biology, or variation in *Cardinium* thermotolerance. Additional research testing the effect of temperature and humidity on *Cardinium* symbioses is essential for understanding how *Cardinium* symbioses more generally respond to stressful environmental conditions.

## Genomic, transcriptomic, and proteomic insights into *Cardinium* physiology and ecology

The increasing accessibility of genome-scale sequencing and analysis has contributed to an increase in published *Cardinium* genomes. Despite the diversity of *Cardinium* symbioses, genetic information is only available for limited host groups. Even more sparse is information on *Cardinium* function at the mRNA and protein level, with only two published *Cardinium* gene transcription datasets (Mann et al. 2017, Gardner et al. 2018). Functional data are essential for deducing how *Cardinium* responds to changes in its cellular environment across host development or environmental conditions at the gene, mRNA, and protein levels. Further, molecular data from *Cardinium* systems are very rarely connected to host phenotype, highlighting a need for integrating molecular data with the phenotypic characterization of *Cardinium* strains to allow for comparative analyses of host–microbe interactions (e.g. reproductive manipulation effectors). In the following sections, we describe our current knowledge of *Cardinium* physiology and its interactions with hosts based on available genomic, transcriptomic, and proteomic data. We will also compare this knowledge to current insights from *Wolbachia*, a distantly related but functionally similar symbiont, to provide a relevant frame of reference for *Cardinium* physiology and ecology as a bacterial endosymbiont.

### Structure of *Cardinium* genomes

There are 21 publicly available *Cardinium* genomes (Table 1), 7 of which are closed: *c*Eper1 from the parasitoid wasp *E. suzannae, c*HgTN10 from the nematode *Heterodera glycines, c*Sfur from the planthopper *S. furcifera, c*Oegib-Wal from the spider *Oedothorax gibbosus*, DF from the mite *Dermatophagoides farinae*, icPhiSpin1 from the beetle *Philonthus spinipes*, and idTipUnca1 from the cranefly *T. unca*. The remaining 14 genomes are contig or scaffold-level genome assemblies, containing anywhere from 5 to 307 contigs. Of the 21 available *Cardinium* genomes, only 2 have been linked to a reproductive manipulation phenotype. Most *Cardinium* genome assemblies are fragmented, likely due to challenges during assembly from insufficient sequencing coverage of the symbiont genome due to large amounts of host DNA (Stouthamer et al. 2018) and repetitive DNA regions in the symbiont genome. Similar to genomes of other obligate intracellular endosymbionts, *Cardinium* genomes have reduced size (between 0.9 and 1.5 Mbp), GC content (between 33.5% and 39.0%), and functional potential (encoding around 1000 predicted genes) (McCutcheon and Moran 2012, Moran 2003, Moran et al. 2008, Moran and Bennett 2014, Moran and Plague 2004). Similarly, *Ca*. Amoebophilus asiaticus, an obligate intracellular symbiont of free-living amoebae and closest known relative to *Cardinium*, has a 1.9 Mbp genome with around 1500 genes and 35% GC content (Schmitz-Esser et al. 2010).

**Table 1.** Publicly available *Cardinium* genomes and associated phenotypic and assembly data. Abbreviations: cytoplasmic incompatibility (CI), no data/unknown (n.d.), and Darwin Tree of Life Project (DTLP).

Strain name	Host	Reproductive manipulation	% GC content	Number of contigs	Plasmids	Genome assembly, plasmid size (kb)	GenBank accession	References
*c*BcalN1	Mite—*B. californicus*	n.d.	36.5	307	n.d.	1046	GCA_022810505.1	
*c*BcalN2	Mite—*B. californicus*	n.d.	36.5	275	n.d.	1049	GCA_022810445.1	
*c*ByotB1	Mite—*Brevipalpus yothersi*	n.d.	36.5	216	n.d.	1086	GCA_022763005.1	
*c*ByotN1	Mite—*B. yothersi*	n.d.	36.5	292	n.d.	1145	GCA_022810525.1	
TP	Mite—*Tyrophagus putrescentiae*	n.d.	39	33	n.d.	914.8	GCA_025215055.1	Xiong et al. (2023)
JH06282024_1	Mite—*T. putrescentiae*	n.d.	39	28	n.d.	1052	GCA_036541165.1	
JH06282024_2	Mite—*T. putrescentiae*	n.d.	38.5	59	n.d.	1090	GCA_036541185.1	
JH06282023_2	Mite—*T. putrescentiae*	n.d.	39	55	n.d.	1052	GCA_030441515.1	
*c*Dfar	Mite—*D. farinae*	n.d.	37	5	n.d.	1482	GCA_007559345.1	Erban et al. (2020)
DF	Mite—*D. farinae*	n.d.	38	1	2	1419, 33.8, and 125.3	GCA_025268635.1	Xiong et al. (2023)
*c*Oegib-Wal	Spider—*O. gibbosus*	n.d.	36.5	1	0	1137	GCA_936981045.1	Halter et al. (2023)
*c*BtQ1	Whitefly—*B. tabaci*	n.d.	36	11	1	1012 and 52.0	GCA_000689375.1	Santos-Garcia et al. (2014)
CanCar	Whitefly—*B. tabaci*	n.d.	36	50	n.d.	996.8	GCA_004300865.1	
*c*Sfur	Planthopper—*S. furcifera*	CI	39.2	1	0	1103	GCA_003351905.1	Zeng et al. (2018)
*c*Eper1	Wasp—*E. suzannae*	CI	36.5	1	1	944.9 and 57.8	GCA_000304455.1	Penz et al. (2012)
icPhiSpin1	Beetle—*P. spinipes*	n.d.	39	1	0	1093	GCA_964030745.1	DTLP
idTipUnca1	Cranefly—*T. unca*	n.d.	36.5	1	0	1374	GCA_964020025.1	DTLP
*c*Cpun	Biting midge—*C. punctatus*	n.d.	33.5	63	0	1137	GCA_004354815.1	Siozios et al. (2019)
*c*HgTN10	Nematode—*H. glycines*	n.d.	38	1	0	1193	GCA_003176915.1	Showmaker et al. (2018)
*c*Ppe	Nematode—*P. penetrans*	n.d.	35.5	45	0	1358	GCA_003788695.1	Brown et al. (2018)
*c*Hhum	Nematode—*Heterodera humuli*	n.d.	38.5	166	0	1056	GCA_028766965.1	Tarlachkov et al. (2023)

Another feature among some endosymbiont genomes undergoing reduction is an increased abundance of mobile genetic elements (Naito and Pawlowska 2016). This also seems to be true for *Cardinium*. For example, transposable elements represent 12.4% of coding sequences (CDS) in *Cardinium c*Eper1 and ~30% of the *c*Oegib-Wal genome (Halter et al. 2023, Penz et al. 2012). In contrast, prophages, which are present in *Wolbachia* genomes, seem to be largely absent in available *Cardinium* genomes (Brown et al. 2018, Halter et al. 2023, Penz et al. 2012). Plasmids appear to be common, although the true prevalence of plasmids in *Cardinium* is difficult to ascertain due to the limited number of closed genomes. Of the seven closed genomes, strains *c*Eper1 and DF were found to harbor one and two plasmids, respectively, and one draft genome (*c*BtQ1) was predicted to have a plasmid. However, plasmid presence needs to be verified for draft assemblies. *Cardinium* plasmids range in size from 33.8 to 125.3 kb and have a reduced % GC content compared to chromosomal DNA. Commonly encoded features on known *Cardinium* plasmids include a plasmid partitioning protein (e.g. ParA), a homolog to a conjugation/recombination enzyme (e.g. TraG), many genes common in smaller mobile genetic elements (e.g. transposases), and many genes with unknown functions. While the functions of *Cardinium* plasmids are currently unknown (hence their status as “cryptic” plasmids), it has been hypothesized that they contain genes important for host interaction (Penz et al. 2012, Santos-Garcia et al. 2014, Xiong et al. 2023).

Despite similarity between *Cardinium* genomes in overall genomic properties, these genomes are highly variable at the gene and nucleotide levels, with a wide range in the average nucleotide identity from around 70% to >99% between genome pairs (Table S2). For example, our analysis using OrthoVenn3 (Sun et al. 2023) on 11 *Cardinium* genomes from various hosts (*c*Eper1, *c*BtQ1, *c*Sfur, *c*Oegib-Wal, DF, TP, icPhiSpin1, idTipUnca1, *c*Cpun, *c*Ppe, and *c*HgTN10) revealed a core set of 389 gene clusters out of the ~1000 genes encoded by a *Cardinium* genome (Fig. S1). Conserved functions (inferred via GO terms of orthologous clusters) are mainly those essential for bacterial life, including DNA replication and repair, transcription, translation, cell homeostasis, protein folding and transport, ATP synthesis, and a limited set of biosynthesis pathways such as those for peptidoglycan and liposaccharides.

### *Cardinium* metabolism and physiology

**Figure 6.**
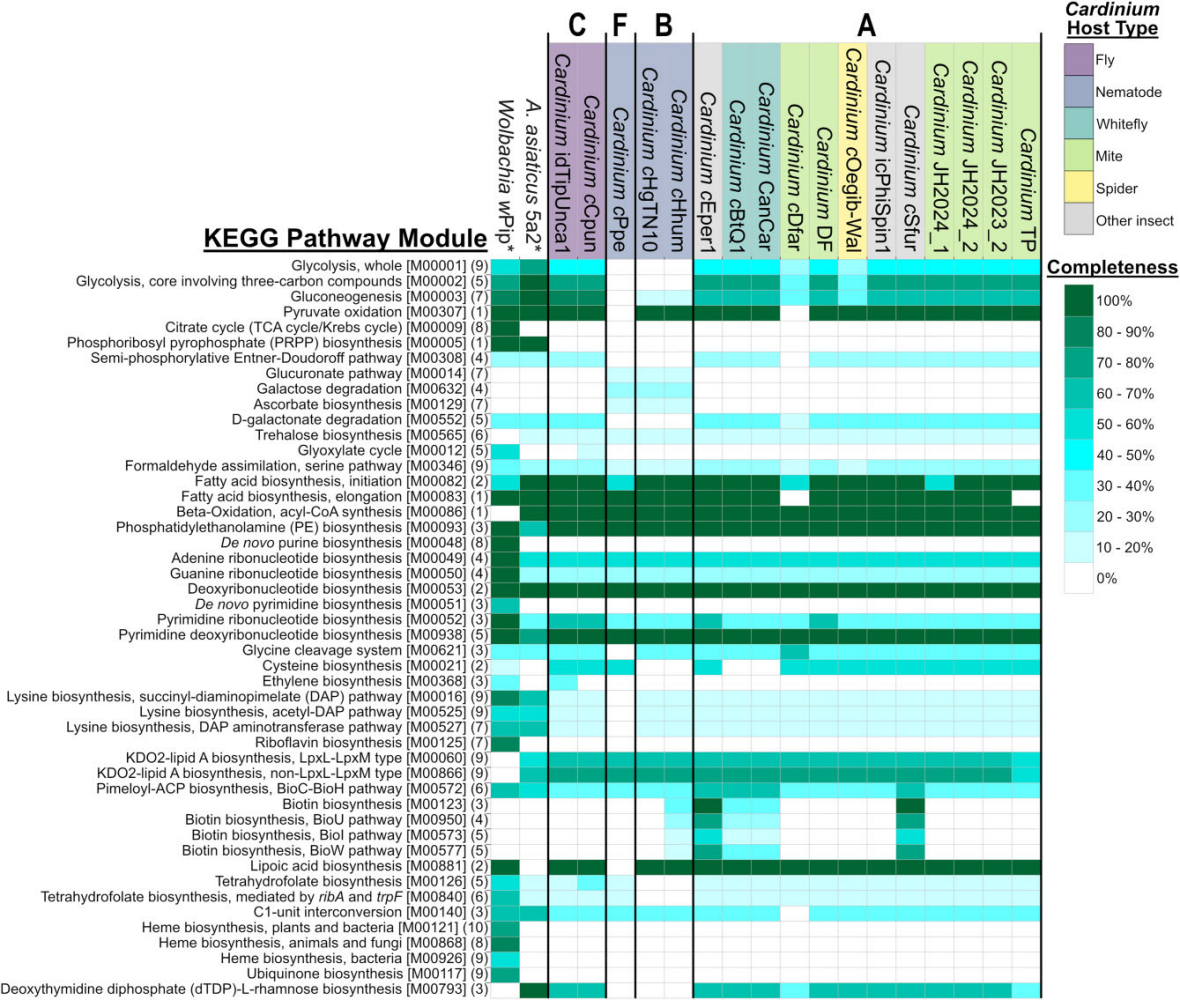
Heatmap comparing the metabolic potential of *Cardinium* genomes to their sister lineage *A. asiaticus* and to *Wolbachia w*Pip. *Wolbachia w*Pip represents a second endosymbiont lineage that shares multiple general features with *Cardinium*, including occupying a similar intracellular niche, commonly occurring in a range of invertebrate hosts, and causing a variety of manipulative and beneficial host phenotypes. A general assessment of the metabolic potential of the genomes was generated using the GhostKOALA service from KEGG (Kanehisa et al. 2016) by combining results from the “module” tab of the KEGG Mapper Reconstruction output for each genome into a heatmap using JColorGrid (Joachimiak et al. 2006). Numbers in parentheses at the end of each pathway name indicate the total number of genes in that pathway, and the accession for each KEGG pathway module is given in brackets. Further, bolded letters above the tree indicate proposed *Cardinium* clades from Fig. 2 and asterisks indicate genomes that have additional pathways not shown in this heatmap, since only select pathways present in *Wolbachia* or *Amoebophilus* but absent in *Cardinium* genomes were included in the figure. Note that high fragmentation of genome assemblies may cause inaccuracies in assessing presence or absence of pathways so functional capacity might be underestimated. Refer to Table 1 for accession numbers and other information regarding *Cardinium* genomes included in this figure. GenBank accession numbers for *A. asiaticus* and *Wolbachia w*Pip are GCA_000020565.1 and GCA_000073005.1.

Due to its obligate intracellular lifestyle and overall limited encoded metabolic capacity, it is difficult to infer the core metabolism and energy generation mechanisms of *Cardinium*. We compiled results from the KEGG GhostKOALA service (Kanehisa et al. 2016) on all available genomes to provide a general overview of *Cardinium* metabolism for this review (Fig. 6).

*Cardinium* has limited metabolic capacity like other obligate intracellular symbionts, with the highest biosynthetic potential occurring in the CI-causing *Cardinium* strains *c*Eper1 and *c*Sfur, while the nematode-associated (*c*Ppe, *c*Hhum, and *c*HgTN10) and mite-associated (*c*Dfar) *Cardinium* strains have lower biosynthetic potential (Fig. 6).

Even highly conserved pathways of central metabolism are reduced or lost entirely in *Cardinium*. This suggests that *Cardinium* relies on many host-derived metabolites and pathway intermediates to make up for its incomplete biosynthetic pathways. Our analysis shows most *Cardinium* genomes encode a partial glycolysis pathway, where only the reactions involving 3-carbon compounds are retained (Fig. 6). Glycolysis is further reduced in *c*Dfar and *c*Oegib-Wal and is completely absent in nematode-associated *Cardinium* (*c*HgTN10, *c*Ppe, and *c*Hhum). This suggests even greater host dependence in these strains. Gluconeogenesis is also incomplete in *Cardinium* and 6-carbon sugars cannot be formed by most strains. This pathway is further reduced in *c*Dfar, *c*Oegib-Wal, and nematode-associated *Cardinium*. The oxidation of pyruvate to acetyl-CoA via pyruvate dehydrogenase is conserved (except for *c*Dfar and *c*Ppe). Additionally, the biosynthesis of lipoate, which is an important cofactor for many enzymes (e.g. pyruvate dehydrogenase) (Spalding and Prigge 2010), is present in all *Cardinium* except *c*Ppe. However, the tricarboxylic acid (TCA) cycle and other central metabolism pathways such as the Entner–Doudoroff pathway and the pentose phosphate pathway appear to be absent in *Cardinium* (Fig. 6). All subunits of adenosine triphosphate (ATP) synthase are also encoded in nearly all *Cardinium* genomes based on BLASTp comparisons, so the ability to generate ATP via a proton gradient across the cell membrane has likely been retained. However, no other components of an electron transport chain are present (Penz et al. 2012). Only some pathways that use intermediates of central carbon metabolism are present. For example, most *Cardinium* are capable of both fatty acid biosynthesis initiation and elongation; however, no *Cardinium* strain can synthesize nucleotides *de novo* and there are very few complete biosynthesis pathways for amino acids, even with *Cardinium* genomes commonly encoding tRNAs for all 20 amino acids (Fig. 6). DNA maintenance also appears to be well-conserved in *Cardinium* with ~42 DNA repair and recombination genes per genome predicted by GhostKOALA (Kanehisa et al. 2016). This includes RecA, which seems to be conserved in all sequenced genomes, likely enabling homologous recombination (Penz et al. 2012).

*Cardinium* encodes pathways for core elements of the bacterial cell membrane, retaining the entire pathway for the synthesis of phosphatidylethanolamine (Fig. 6), a core lipid of bacterial cell membranes (Murzyn et al. 2005), and most of the pathway for producing peptidoglycan based on amino acid similarities to known peptidoglycan biosynthesis proteins. *Cardinium* has also retained its ability to produce some components of lipopolysaccharide (LPS), an outer membrane feature characteristic of Gram-negative bacteria. For example, most of the genes required for the biosynthesis of lipid A, which causes the endotoxic effects of LPS (Gronow and Brade 2001), are conserved across all *Cardinium*. In addition, the production pathway of dTDP-l-rhamnose, a precursor to rhamnose and prominent component of the O-antigen of LPS (Tsukioka et al. 1997), is also mostly complete in all *Cardinium* other than those associated with nematodes (*c*HgTN10, *c*Ppe, and *c*Hhum) (Fig. 6). It has been proposed that the presence of LPS in the *Cardinium* outer membrane may cause it to induce a stronger immune response in some hosts, thus potentially limiting its host range compared to *Wolbachia*, which has lost most of the genes required for LPS biosynthesis (Weinert et al. 2015). Future work incorporating expression data will discern the relevance of these cell surface features, which will be important for evaluating this hypothesis.

*Cardinium* strains *c*Eper1 and *c*Sfur encode a complete biotin synthesis pathway, but this pathway is only partially encoded by *c*BtQ1, CanCar, and *c*Hhum, and completely absent in *A. asiaticus* and all other sequenced *Cardinium* strains (Fig. 6) (Brown et al. 2018, Penz et al. 2012, Santos-Garcia et al. 2014, Siozios et al. 2019, Zeng et al. 2018). The degradation or absence of the biotin synthesis pathway in most *Cardinium* suggests that supplementation of this nutrient is not needed for the bacterium itself, nor is it a key component of most *Cardinium*–host symbioses. It is possible the biotin synthesis capabilities of *c*Eper1 and *c*Sfur provide a nutritional benefit to their hosts, as B-vitamins are broadly important for arthropod fitness but are absent in specialized diets like blood and phloem (Serrato-Salas and Gendrin 2023). Therefore, the supplementation of B-vitamins by endosymbiotic bacteria, such as *Wolbachia*, to their hosts can play an important role in nutritional symbiosis (Ju et al. 2020). A role in biotin provisioning is plausible for *c*Sfur, which is hosted by a sap-feeding planthopper. However, it seems unlikely for *c*Eper1 to provide a large nutritional benefit through biotin provisioning as its host is a parasitoid that presumably acquires biotin from its whitefly diet. Evidence points toward a potential horizontal transfer of the core biotin synthesis gene cluster encoded by *c*Eper1 and *c*Sfur (*bioA, bioD, bioC, bioH, bioF*, and *bioB*) between *Cardinium* and *Wolbachia*, as these genes occur in the same arrangement in *Cardinium* and *Wolbachia* and have greater sequence similarity to *Wolbachia*-encoded homologs than to genes encoded by other bacteria in the order *Cytophagales* (phylum: *Bacteroidota*) (Nikoh et al. 2014, Penz et al. 2012, Zeng et al. 2018). Overall, much more remains to be explored regarding the role, origins, and retention of the biotin synthesis pathway in *Cardinium*.

*Cardinium* has a greatly reduced biosynthetic potential compared to *Wolbachia*. Relative to *Cardinium, Wolbachia* strain *w*Pip is enriched in KEGG functional categories for the metabolism of carbohydrates, nucleotides, cofactors, vitamins, lipids, and amino acids. *Wolbachia* encodes the entire TCA cycle, most of the nonoxidative pentose phosphate pathway, and can synthesize phosphoribosyl pyrophosphate, nucleotides, and glutathione, all of which are mostly absent in *Cardinium. Wolbachia* also has nearly complete pathways for the biosynthesis of lysine, riboflavin, heme, ubiquinone, and tetrahydrofolate (Fig. 6). The only biosynthetic pathways, which appear to be more complete in *Cardinium* than *Wolbachia w*Pip are those involved in LPS production and biotin synthesis, the latter of which is complete in only two *Cardinium* genomes (Fig. 6). Overall, the potential for *Cardinium* to provision nutrients to its hosts appears to be less than that of *Wolbachia*; however, additional genomes will provide further context for the full extent of *Cardinium* metabolism.

### Genome-encoded features for host interaction

While lacking in biosynthetic potential, *Cardinium* genomes are rich in predicted eukaryotic-interacting proteins (e.g. proteins with ankyrin repeats, tetratricopeptide repeats, leucine-rich repeats, and F- and U-box domains; Frank 2019, Martyn et al. 2022), as well as transport proteins and secretion systems. These features may be involved in interactions ranging from host immune evasion to reproductive manipulation and are likely integral to the host-associated lifestyle of *Cardinium*. However, much is still unclear regarding how *Cardinium* uses these genes for interactions with its hosts, and, as such, they are important targets for characterizing how *Cardinium* interfaces with and influences a wide range of host organisms.

Due to its drastically reduced biosynthetic capacity, *Cardinium* likely relies heavily on its transporters to obtain necessary host-derived metabolites, nucleotides, and other molecules. Most *Cardinium* genomes encode ~38 transport proteins as predicted by GhostKOALA (∼4% of CDS), but this number is likely an underestimate based on reported numbers from previously published genomes [60 predicted transporters in *c*Eper1 and 80 in *c*Sfur (7.1% and 10% of CDS, respectively) (Penz et al. 2012, Zeng et al. 2018)]. These are predicted to include oligopeptide transporters, dicarboxylate transporters, an ATP/ADP (adenosine diphosphate) antiporter, and other putative nucleotide transporters, among others, suggesting that *Cardinium* can import many essential compounds from its surrounding host cell (Penz et al. 2012, Zeng et al. 2018).

*Cardinium* also encodes multiple secretion systems for exporting synthesized effectors and other proteins to host cells. All *Cardinium* sequenced, thus far have both the sec-dependent secretion pathway for transporting unfolded proteins across the cytoplasmic membrane and homologs of the novel phage-derived type VI secretion system (T6SS^iv^) characterized in *A. asiaticus* (Böck et al. 2017) based on gene annotations and amino acid similarities to known proteins. The intracellular components of the T6SS^iv^ likely form the prominent columns for which *Cardinium* is named and which are a key characteristic of its cellular ultrastructure (Fig. 1; Böck et al. 2017). The effector proteins secreted by this secretion system are unknown, but the prominence and high transcription level of the assembled T6SS^iv^ within *Cardinium* cells (Mann et al. 2017) and prevalence of this secretion system across the entire lineage suggest it plays an important role in protein secretion and/or interaction with hosts or other microbes. MacSyFinder (Néron et al. 2023) also predicts that some *Cardinium* strains additionally encode a type I secretion system (T1SS), including CanCar, *c*BtQ1, *c*Cpun, *c*Dfar, DF, icPhiSpin1, and TP. Further research is required to confirm whether the T1SS is assembled and functional in these *Cardinium* strains and what its role in cell physiology may be, as many sequenced *Cardinium* appear to lack T1SS component homologs.

Some *Cardinium* genomes, including *c*Cpun, *c*BtQ1, *c*Sfur, *c*Oegib-Wal, icPhiSpin1, and idTipUnca1, encode at least four homologs to genes relating to gliding motility (such as *gldK, gldL, gldM*, and *gldN*) (Halter et al. 2023, Santos-Garcia et al. 2014, Siozios et al. 2019, Zeng et al. 2018). The role of these *Cardinium* genes in gliding motility is debatable, however, since many core genes are missing and *Cardinium* strains like *c*Ppe that lack this machinery can be found throughout the body of their host (Brown et al. 2018). It has been posited that these four proteins could instead be involved in protein secretion via a role in building the type 9 secretion system (T9SS) common to Bacteroidota (McBride 2019). However, it should be noted that many of the genes required for the T9SS are absent. An alternative hypothesis speculates that the T9SS may be ancestral to *Cardinium* and may have undergone gradual loss following replacement with the T6SS^iv^ as the dominant protein secretion system in *Cardinium*. This idea has yet to be examined (Siozios et al. 2019), and more research is required to identify the significance and evolutionary origins of gliding motility proteins in *Cardinium*.

Finally, the recent increase in availability of *Cardinium* genomes and the identification of factors involved in reproductive manipulations caused by other symbionts provides an opportunity for expanded comparative analyses identifying the presence, or lack thereof, of homologous manipulation factors within *Cardinium* genomes. The initial comparative analysis between CI-inducing *c*Eper1 and CI *Wolbachia* strains found little homology between the distantly related bacteria (Penz et al. 2012). Since that study, factors involved in *Wolbachia* CI have been identified, with a more recent comparative analysis confirming that *Cardinium* genomes do not encode the factors responsible for *Wolbachia* CI, and thus acquired its CI manipulation independently (Lindsey et al. 2018). Unfortunately, the absence of genomes for feminizing and parthenogenesis-inducing *Cardinium* precludes a comparative analysis of effector genes, like the recently identified factors implicated in *Wolbachia* PI, at this time (Fricke and Lindsey 2024b, Li et al. 2024b). Comparative genomics studies comparing phenotypically similar *Cardinium* symbionts would provide insights into potential *Cardinium* reproductive manipulation factors.

### *Cardinium* transcriptomics

To date, there are two published studies on transcription levels of *Cardinium*. The first *Cardinium* transcriptome was published by Mann et al. (2017) comparing transcription levels between *Cardinium c*Eper1 from male and female adult *E. suzannae* to identify potential CI candidate genes. A second study, published by Gardner et al. (2018), assembled 468 *Cardinium* transcripts from the nematode, *H. glycines*, and provided a brief Gene Ontology (GO) overview. Both studies found housekeeping genes (i.e. genes involved in cell maintenance like DNA maintenance, transcription, and translation) and transporters to be transcribed at the highest overall levels (Mann et al. 2017, Gardner et al. 2018). Below, we summarize the unique features of the strains presented in these two studies.

Most of what is known about *Cardinium* transcription is based on findings in the *c*Eper1 strain. Mann et al. (2017) found biotin synthase was highly transcribed, along with other genes in the biotin synthesis pathway, which were moderately transcribed. As discussed above, the benefit of biotin for *c*Eper1 or *E. suzannae* is unclear as *E. suzannae* should obtain B-vitamins from their larval whitefly diet. Additionally, 55.8% of the 129-predicted transposases within the *Cardinium c*Eper1 genome were transcribed, although it is unclear whether they are active in transposition (Mann et al. 2017). Finally, there were also many highly transcribed hypothetical proteins with no known or predicted function, some of which were classified as candidates for causing CI based on predicted protein domains, annotations, and gene expression levels (Mann et al. 2017). Although *Cardinium* transcription does not differ greatly based on host sex generally, there were 15 differentially transcribed *Cardinium* genes between male and female hosts, with three upregulated in *c*Eper1 infecting male hosts and 12 upregulated in *c*Eper1 infecting female hosts (Mann et al. 2017). Five of the *Cardinium* genes upregulated in female hosts encode ribosomal proteins, one encodes a tRNA ligase, and two encode RNA polymerase subunits, suggesting an overall increase in transcription and translation by *c*Eper1 when infecting female *E. suzannae*. Only one *c*Eper1 gene upregulated in females is an uncharacterized hypothetical protein, while all three *Cardinium* genes upregulated in males are uncharacterized, with two being adjacent plasmid-encoded genes. It is possible that more pronounced differences may be found among *Cardinium* infecting different sexes of other hosts, *Encarsia* of earlier life stages, or when analyzing tissue/organ-specific gene expression patterns.

Gardner et al. (2018) applied transcriptomics to identify host cell manipulation effectors from the early life stages of the soybean cyst nematode *H. glycines* and, as a by-product, assembled 468 *Cardinium* transcripts from infected nematodes. Subsequent analysis of GO terms revealed that the most abundant biological processes assigned to *Cardinium* transcripts in this host were mainly involved in cell maintenance and growth: translation (14%), transport (10%), DNA metabolic processes (8%), and carboxylic acid metabolic processes (8%) (Gardner et al. 2018). The main molecular functions assigned to *Cardinium* transcripts included ATP binding (24%), DNA binding (17%), RNA binding (14%), and metal ion binding (11%), suggesting that these features may play key roles in the association of *Cardinium* with *H. glycines* (Gardner et al. 2018).

Overall, our knowledge on *Cardinium* gene expression at the transcript level remains limited, with only two strains represented. Additional published transcriptomic studies of *Cardinium* hosts in which host gene expression was the focus (Abbà et al. 2022, Li et al. 2020a, Liu et al. 2023, Yang et al. 2021) could be leveraged, along with future transcriptomic and proteomic data, for a more comprehensive overview of regulatory, functional, and host interaction features associated with *Cardinium*.

### Host responses to *Cardinium* infection

Understanding how a variety of hosts respond to infection by *Cardinium* is important for developing a general understanding of *Cardinium—*host interactions. Yet, our knowledge on host transcriptomic and proteomic changes in response to *Cardinium* largely derives from two hosts: the house dust mite, *D. farinae*, and MED *Bemisia* whiteflies (also called *B. tabaci* Biotype Q).

In a transcriptome study of *D. farinae, Cardinium* infection was negatively correlated with the expression of several host metabolic pathways, such as glycolysis and the TCA cycle (Hubert et al. 2021a), suggesting that *Cardinium* infection may decrease rates of *D. farinae* central metabolism. Hubert et al. (2021a) also hypothesized that, since *Cardinium* gene transcription was correlated with the transcription levels of mite genes involved in terpenoid backbone biosynthesis, *Cardinium* may stimulate the production of mite pheromones involved in reproduction or aggregation, which could potentially enhance the transmission of the maternally inherited symbiont. Additionally, the expression of some *Cardinium* genes was correlated with the transcription of various mite immune system genes, suggesting that *Cardinium* may influence the immune system of its mite host, perhaps through activation of mite Toll-like receptors or by altering the transcription level of caspases, which induce apoptosis of host cells (Hubert et al. 2021a). An additional study found that *D. farinae* mite exosomes, which are small mite-secreted organelles implicated in human allergic respiratory disease due to the molecules they carry as cargo, contain *Cardinium*-derived proteins: 17 out of 72 proteins detected in exosomes were *Cardinium* proteins (Yang et al. 2023b). This suggests *Cardinium* may also play a role in the allergenicity of the house dust mite (Yang et al. 2023b).

*Cardinium* has been linked to a variety of changes in MED *Bemisia* gene expression, which may contribute to changes in host phenotypes. For example, *Cardinium* infection can upregulate host genes involved in cellular homeostasis, metamorphosis, autophagy, and the ubiquitin/proteasome system, all of which may be involved in the increased thermotolerance of some *Cardinium*-infected whiteflies (Yang et al. 2021). *Cardinium* is also associated with decreased transcription of some whitefly detoxification genes (Liu et al. 2023). This change in detoxification gene transcription may be in part a response to altered host plant transcription, as cotton plants in this study also exhibited a downregulation of defense response genes when the plants were infested with *Cardinium*-infected whiteflies versus uninfected whiteflies (Liu et al. 2023). It is possible that *Cardinium* may mediate whitefly–plant interactions by suppressing plant antiherbivory defenses.

The only example of host responses to *Cardinium* at the protein level also focused on uninfected and *Cardinium*-infected MED *Bemisia* whiteflies (Li et al. 2018a). In that study, there were 146 differentially expressed host proteins in response to *Cardinium* infection. Upregulated proteins may be involved in the whitefly immune response, as they were associated with RNA metabolism and pathways involved in signaling, drug resistance, apoptosis, and the metabolism of glyoxylate and dicarboxylate. The metabolism of retinol, a vitamin crucial for many aspects of host growth, development, and immune function was also upregulated (Li et al. 2024a, Morriss-Kay and Wardt 1999). Downregulated genes were enriched in functions involved in DNA processing and the spliceosome, suggesting that host pre-mRNA splicing may be altered during *Cardinium* infection (Li et al. 2018a). A later study by the same group found that *Cardinium* infection induced 23 differentially expressed MED *Bemisia* microRNAs (miRNAs), which posttranscriptionally regulate genes via prevention of mRNA translation (Bartel 2004, Li et al. 2018b). These *Cardinium*-responsive miRNAs may be responsible for some outcomes seen via proteomics, as they are predicted to regulate genes involved in female host development, immune response, and energy metabolism, as well as inhibit apoptosis and decrease host resistance to thermal stress and insecticides during *Cardinium* infection (Li et al. 2018b).

Overall, *Cardinium* and host gene expression analyses are severely lacking and largely limited to a single host species. New experiments designed to interrogate specific questions (e.g. *Cardinium* gene expression differences in different host sexes, life stages, organs, and so on) are essential to understand the mechanisms of *Cardinium*–host interactions. Furthermore, additional datasets and analysis of *Cardinium* infecting various hosts with confirmed phenotypes (e.g. CI, PI, increased fecundity, and so on) would be an asset to emerging *Cardinium* research.

## Present and future directions in *Cardinium* research

**Figure 7.**
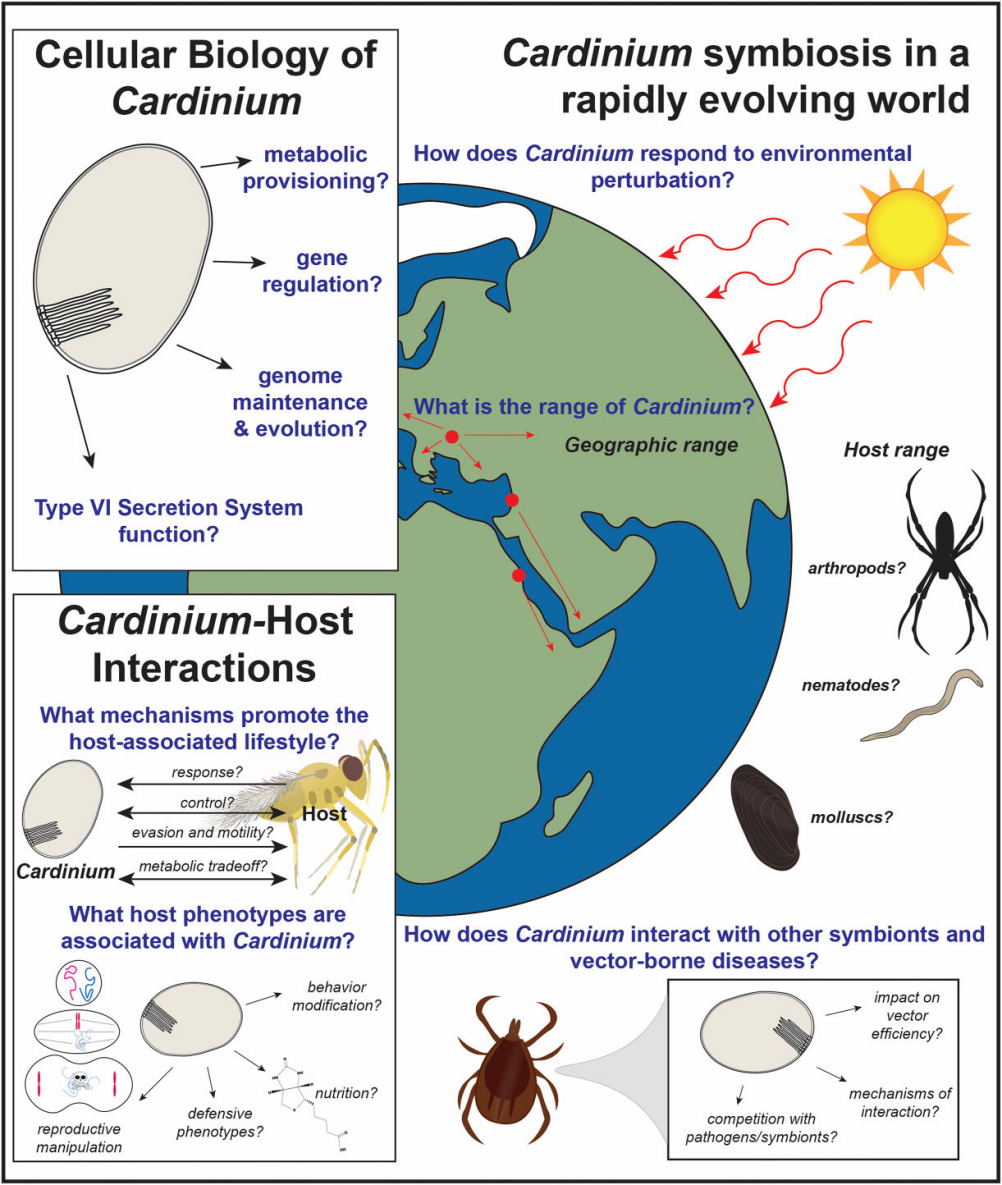
Future directions and open questions in *Cardinium* research. These directions include researching *Cardinium* cellular biology and *Cardinium*–host interactions to better understand how this symbiont modifies host biology. Investigations into *Cardinium* interactions with co-infecting microbes, particularly pathogens, could provide a basis for incorporating *Cardinium* into arthropod pest control strategies. Expanded characterizations of *Cardinium* host range and the influence of environmental factors on *Cardinium* infection dynamics may help predict how climate shifts impact widespread heritable endosymbionts like *Cardinium* and their invertebrate hosts.

Substantial progress has been made over the past 30 years in our understanding of the widespread invertebrate endosymbiont, *C. hertigii*. Research has shown *Cardinium* to be a symbiont of numerous invertebrate lineages, capable of manipulating host biology in a variety of ways. The sequencing of *Cardinium* genomes provided additional insights into the biology of this endosymbiont, such as revealing its limited metabolic potential, identifying a novel phage-derived Type VI secretion system, and confirming an independent origin of *Cardinium* CI. Still, gaps in our understanding of *Cardinium* remain, leaving many important areas of *Cardinium* biology open for exploration, such as the general cellular biology of *Cardinium*, the host–microbe interactions of *Cardinium* symbioses on molecular and phenotypic scales, and the global dynamics of the broader ecophysiology and evolution of *Cardinium* (Fig. 7). These areas of research will be critical to address as we start to consider potential applications for *Cardinium* in agricultural and human health contexts.

The increased accessibility of genomic sequencing has been a boon for symbiosis research broadly, generating insights into the molecular biology and evolution of uncultivable bacterial symbionts like *Cardinium*. For example, several *Cardinium* genomes have been published over the last decade, providing information on the functional potential of these symbionts. Yet, studies exploring *Cardinium* gene expression at the transcript and protein levels remain limited. Such analyses are essential for learning how *Cardinium* survives in and responds to its host environment. Proteomic analyses are especially needed, as they have played a critical role in the identification of candidate factors involved in symbiont physiology and host interaction, including the *Wolbachia* CI factors (Beckmann and Fallon 2013, Beckmann et al. 2017, LePage et al. 2017). Furthermore, the increasing availability of more advanced sequencing techniques and expression analyses, such as single-cell transcriptomics and proteomics, may further facilitate research into how *Cardinium* responds to and modifies its native host cell environment.

To date, *Cardinium–*host interaction research has largely focused on the phenotypic consequences of infection for animal hosts; however, information on the molecular characteristics and products of *Cardinium* that drive these phenotypes remains limited. For example, an understanding of the molecular mechanisms underlying *Cardinium* reproductive manipulation is currently lacking. While research on *Encarsia* wasps has provided new details on *Cardinium* CI modification and its resulting cytological defects, it remains to be seen what molecular features induce these drastic phenotypes. Gaining a general understanding of how *Cardinium* manipulates host biology will require mechanistic information from a variety of host systems. The recent advances in the accessibility of genome sequencing and gene expression analyses, coupled with the use of heterologous expression systems in model organisms like *Saccharomyces cerevisiae*, offer an opportunity to unravel the molecular mechanisms underlying heritable symbioses even in less tractable host systems (Murphy and Beckmann 2024).

Our understanding of *Cardinium* host effects beyond reproductive manipulation is even more limited. In particular, studies exploring interactions between *Cardinium* and host pathogens are surprisingly lacking given symbiont-mediated protection and potential for disease mitigation granted by other common symbionts (O’Neill et al. 2018, Ballinger and Perlman 2019, Gong et al. 2023). Research into how *Cardinium* modifies the vector potential of important crop pests like planthoppers or animal disease vectors, such as biting midges will be integral to understanding the potential of this symbiont as a tool for arthropod-vectored disease control (Mellor et al. 2000, Morag et al. 2012, Li et al. 2020a). Research into the host benefits granted by *Cardinium* infection will also improve our understanding of *Cardinium*/host interactions, including how this symbiont spreads through host populations, and the evolutionary dynamics of these symbioses.

Direct molecular interrogation of *Cardinium* biology has been challenging due in part to the obligate intracellular lifestyle of the bacterium. Developing tools for direct molecular manipulation of *Cardinium* would open a number of doors for understanding basic aspects of *Cardinium* biology and interactions with its host, ranging from functionally characterizing candidate factors for host manipulation or growth regulation to identifying novel host interaction features important for maintaining symbiosis. For example, peptide nucleic acids (Pelc et al. 2015) and CRISPR-based technologies have seen increasing use in manipulating formerly intractable bacteria, including *Rickettsia* spp. which commonly associate with insects (McClure et al. 2017, Fisher and Beare 2023). Chemical mutagenesis is another intriguing option for manipulating *Cardinium*, as it does not require *Cardinium* cell cultures and could potentially be applied to *Cardinium* living in a natural host (McClure et al. 2017). Using chemical mutagenesis in combination with genetics approaches could enable researchers to generate and identify hosts harboring *Cardinium* mutants with altered phenotypes (McClure et al. 2017, Duarte et al. 2021). Such an approach has been successfully used to identify genes important for regulating *Wolbachia* proliferation (Duarte et al. 2021). The ability to culture *Cardinium* cells in different arthropod cell lines (Kurtti et al. 1996, Morimoto et al. 2006, Nakamura et al. 2011) combined with these emerging methods for molecular manipulation of obligate intracellular bacteria is a promising route to elucidate the molecular mechanisms that underlie *Cardinium–*host interactions.

Just as important as understanding how *Cardinium* interacts with its host, is the range of hosts with which *Cardinium* interacts. Recent surveys suggest that the host range of *Cardinium* is broad, including species across multiple phyla, indicating that *Cardinium* likely acts as an unseen player in the biology of a substantial number of invertebrate species. For example, identification of *Cardinium* in entirely new host groups, like mollusks, suggests that current estimates of *Cardinium* prevalence do not reflect its true global prevalence and host range (Weinert et al. 2015). Clearly, more surveys across host taxa are needed to better understand this symbiont’s host range, particularly in host taxa that have only had limited screening, like nonmarine crustaceans, or taxa belonging to groups commonly harboring *Cardinium*, like spiders. The increasing ease and usage of high throughput sequencing techniques, like 16S rRNA amplicon sequencing, will enable these advancements, making larger surveys that target a broader range of potential hosts feasible.

The effect of geographic and climate variation on *Cardinium* infection dynamics should also be explored. Even with their sustained successful spread across invertebrates, some heritable symbionts, like *Cardinium*, can be highly susceptible to thermal stress. Understanding how these symbionts respond to a climate with increasing and more variable temperatures remains a high priority research topic. Given the fundamental roles that *Cardinium* and other symbionts play in their host’s biology (e.g. influencing sex allocation, offspring survival, and fecundity), the effects of the environment on heritable symbionts will also have wide ranging consequences for their invertebrate hosts. This could also have anthropogenic implications, as symbiont thermal sensitivity may affect the potential application of symbionts in pest management and control of insect-vectored diseases, highlighting the need to better understand the interactions between climate and heritable symbioses. Surveys of natural *Cardinium-*infected host populations, particularly across seasonal or spatial gradients, could provide a more accurate understanding of present day *Cardinium* infection dynamics, while laboratory studies can be designed to better replicate natural thermal conditions of the present day and predicted future climate conditions. Global surveys may also offer a great opportunity to recruit and educate members of the general public and scientific community across the world via citizen science through a program similar to the *Wolbachia* Project and its associated database (Lemon et al. 2020). Together, these types of studies will generate a clearer understanding of global *Cardinium* infection dynamics.

While much remains to be uncovered regarding *Cardinium* evolution, ecology, and molecular physiology, advancements in the fields of bioinformatics and molecular biology offer a wealth of opportunities to learn more about *Cardinium*. The coming years will likely bring new information on how *Cardinium* manipulates host biology, as well as how *Cardinium* and their hosts may respond to a changing climate. This will lay the groundwork for potential applications of *Cardinium* in areas, such as pest management and arthropod-vector disease control and prevention in the not-so-distant future.

## Supplementary Material

fuaf031_Supplemental_Files
